# Differences in Transcriptional Dynamics Between T-cells and Macrophages as Determined by a Three-State Mathematical Model

**DOI:** 10.1038/s41598-020-59008-0

**Published:** 2020-02-10

**Authors:** Catherine DeMarino, Maria Cowen, Michelle L. Pleet, Daniel O. Pinto, Pooja Khatkar, James Erickson, Steffen S. Docken, Nicholas Russell, Blake Reichmuth, Tin Phan, Yang Kuang, Daniel M. Anderson, Maria Emelianenko, Fatah Kashanchi

**Affiliations:** 1 Laboratory of Molecular Virology, School of Systems Biology, George Mason University, Manassas, VA USA; 2 Department of Mathematics, University of California Davis, Davis, CA USA; 3 Department of Mathematical Sciences, University of Delaware, Newark, DE USA; 4 Department of Mathematical Sciences, George Mason University, Fairfax, VA USA; 5 School of Mathematical and Statistical Sciences, Arizona State University, Tempe, AZ USA

**Keywords:** Computational biology and bioinformatics, Retrovirus

## Abstract

HIV-1 viral transcription persists in patients despite antiretroviral treatment, potentially due to intermittent HIV-1 LTR activation. While several mathematical models have been explored in the context of LTR-protein interactions, in this work for the first time HIV-1 LTR model featuring repressed, intermediate, and activated LTR states is integrated with generation of long (*env*) and short (TAR) RNAs and proteins (Tat, Pr55, and p24) in T-cells and macrophages using both cell lines and infected primary cells. This type of extended modeling framework allows us to compare and contrast behavior of these two cell types. We demonstrate that they exhibit unique LTR dynamics, which ultimately results in differences in the magnitude of viral products generated. One of the distinctive features of this work is that it relies on experimental data in reaction rate computations. Two RNA transcription rates from the activated promoter states are fit by comparison of experimental data to model predictions. Fitting to the data also provides estimates for the degradation/exit rates for long and short viral RNA. Our experimentally generated data is in reasonable agreement for the T-cell as well macrophage population and gives strong evidence in support of using the proposed integrated modeling paradigm. Sensitivity analysis performed using Latin hypercube sampling method confirms robustness of the model with respect to small parameter perturbations. Finally, incorporation of a transcription inhibitor (F07#13) into the governing equations demonstrates how the model can be used to assess drug efficacy. Collectively, our model indicates transcriptional differences between latently HIV-1 infected T-cells and macrophages and provides a novel platform to study various transcriptional dynamics leading to latency or activation in numerous cell types and physiological conditions.

## Introduction

Since 1996, the rise of combination antiretroviral therapy (cART) has increased the survival of HIV-1 infected patients and has drastically slowed the transmission of the virus from person to person^1^. The lifelong treatment effectively lowers viral titers to undetectable levels in infected individuals; however, this treatment regimen has several limitations, including the need for strict adherence to prevent a viral rebound, the inability to prevent drug resistance, and low penetration to viral reservoirs in the central nervous system (CNS)^2–4^. More recently, these viral reservoirs have been shown to be transcriptionally active as evident from a number of reports including one study of 190 patients under cART, where low levels of HIV-1 RNA (<50 copies/mL) were found in the blood, however, cell-associated RNA copies were still present at approximately 10^3^ copies/10^6^ CD4^+^ T-cells^5^. These findings were further replicated in cells of the CNS origin where around 10^3^ copies of HIV-1 RNA have been found post-mortem in several regions of the brain from HIV-1 infected individuals under long term cART^6^.

HIV-1 can establish infection by integration of the provirus into the genome of long-lived reservoirs such as memory CD4^+^ T-cells. After integration, which typically takes place within an actively transcribed region of the genome, several HIV-1 proteins are produced at a low rate including the Trans-Activator of Transcription (Tat) protein, which is produced from a doubly spliced transcript. Tat then associates with positive transcriptional elongation factor b (p-TEFb), composed of CDK9 and Cyclin T_1_, in the cytoplasm^7^. This complex can enter the nucleus and activate transcription of the HIV-1 genome by two mechanisms: phosphorylation of Negative Elongation Factor (NELF), and phosphorylation of the CTD of RNA polymerase II (RNAPII). Unphosphorylated NELF binds to the trans-activating response region (TAR), a short stem and loop structure of RNA which is located at the 5′ long terminal repeat (LTR) of the viral genome downstream of the transcription initiation site. Phosphorylation of NELF thereby releases the protein from TAR, alleviating its inhibition of the paused RNAPII^8–11^. Subsequent phosphorylation of the RNAPII CTD leads to activated transcription of the HIV-1 genome, approximately 50–100 fold above basal transcription levels^12–14^.

In the absence of Tat, the LTR of the HIV-1 provirus is primarily inactive, however, it can intermittently transition to an active state, resulting in elongation of HIV-1 transcripts and leading to high stochastic variability in HIV-1 gene expression, termed transcriptional noise^15–27^. Modeling of HIV-1 transcription in T-cells has shown that gene expression occurs by randomly timed bursts of transcriptional activity from the 5′ LTR. Furthermore, each burst of transcriptional activity results in 2–10 mRNA transcripts from the activated promoter before returning to inactive state, causing a reduction in Tat protein levels and resulting in reduced Tat-mediated positive feedback^13,28^. The gene expression noise that arises as a result of HIV-1 Tat gene expression fluctuations promotes HIV-1 viral latency^28,29^. Overall, the variation in Tat expression results in the production of both short (TAR; non-coding) and long RNA (*env*; full length genomic) in different proportions in the presence and absence of Tat^30^. The implications of high proportions of short, non-coding TAR RNA has previously been studied in recipient cells, and results suggest there is activation of the innate immune response through cytokine induction and an increase in viral susceptibility in recipient cells^31–35^.

We have long been interested in defining the biochemical pathways that allow for HIV-1 LTR basal and activated transcription between cell types. In this manuscript, we attempted to detect any alterations in the various LTR states using a combination of *in vitro* biochemical assays and mathematical modeling. Our rationale for performing these experiments was that most published literature on HIV-1 transcription modeling utilizes data from several publications which come from numerous sources and use various different cell types to score for basal and activated transcription. Furthermore, these models have not compared potential differences due to cell type when it comes to T-cells *vs*. myeloid transcription, nor have they looked at multiple RNA transcripts^27–29^. To address this, we have analyzed transcription and subsequent production of viral components in HIV-1 infected myeloid and T-cells using biochemical assays to construct parameter values for a novel mathematical model. Furthermore, we have incorporated the use of a transcription inhibitor into our model to analyze cell-specific responses to treatment in terms of LTR activation and the associated RNA and protein production. Here, we present a mathematical model which can be used to analyze changes in transcriptional activation. Our results show a dynamic change of various LTR states that varies between cell types, specifically in terms of activation timing and magnitude of viral component production. Furthermore, our data suggests there may be distinct differences in viral latency amongst cell types which could have broad implications in the treatment of HIV-1 infected patients.

## Results

### Dynamics of LTR activation in T-cells

**Figure 1:**
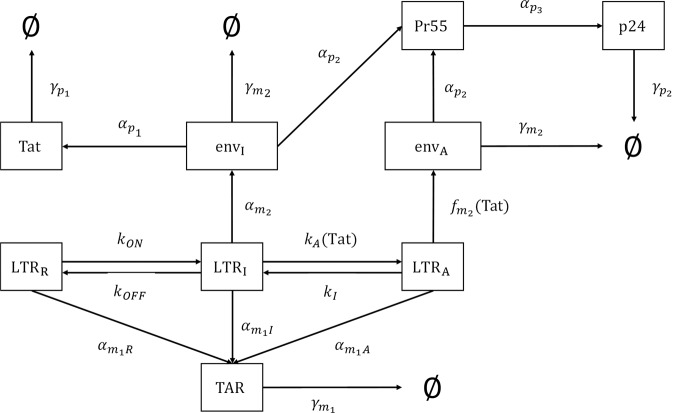
**Schematic diagram** **of a three-state mathematical** **model of HIV-1** **transcription**. The HIV-1 LTR is represented in three states of activation (as opposed to a two-stage model where activation by noise and its regulation in Tat activity is not considered); LTR_*R*_ denotes a repressed state (i.e. latency); LTR_*I*_ represents an intermediate state of activation; and LTR_*A*_ is a Tat-dependent activated state of the HIV-1 LTR in which full viral production is possible. The terms *k*_*ON*_ and *k*_*OFF*_ represent the rate of activation from latency and the return to latency, respectively. *K*_*A*_(Tat) is the rate of Tat-dependent activation from an intermediate LTR to a fully activated LTR state and the term *k*_*I*_ represents the rate in the opposite direction. The diagram depicts the creation of two species of HIV-1 RNAs termed TAR and *env* (envelope). The rate at which TAR RNA is created is given by α_*m1R*_, α_*m1I*_ and, α_*m1A*_ and the TAR degradation/exportation rate is denoted by γ_*m1*_. The HIV-1 RNA species *env* (genomic) is produced by the intermediate state LTR (*env*_*I*_) at a rate of α_*m2I*_. HIV-1 Tat results in a positive feedback mechanism for virus replication, production at a rate of α_*p1*_ and Pr55 (Gag) production at a rate of α_*p2*_. The activated state of the LTR also produces envelope, termed *env*_*A*_, at a Tat-dependent rate of *f*_*m2*_(Tat). The Tat-dependent production of env_*A*_ also results in the production of Pr55 (Gag) at a rate α_*p2*_. Finally, this model depicts the rate cleavage of Pr55 (Gag) to the viral protein p24 via α_*p3*_. Degradation rates (γ_*p1*_, γ_*p2*_, γ_*m1*_, γ_*m2*_) were set to zero for preliminary results.

**Figure 2:**
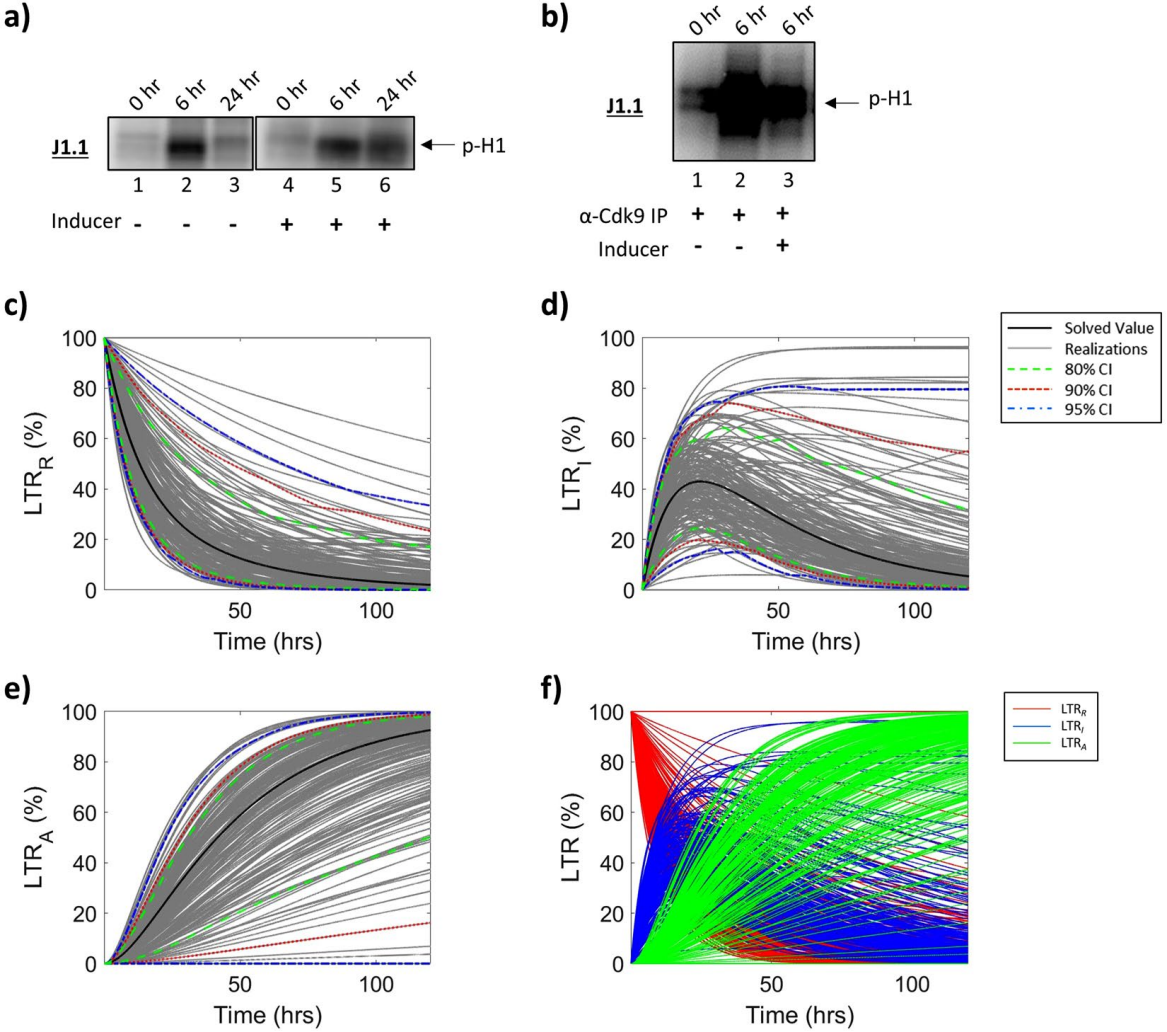
**HIV-1 LTR Dynamics in T-cells**. J1.1 (HIV-1 infected T-cells) were cultured in low serum media (0.1% FBS) for 36 h, and subsequently incubated in 20% FBS media and treated with an inducer (IR). Resulting whole cell extracts were analyzed using an *in vitro* total kinase assay (**a**) or a CDK9 IP kinase assay (**b**) to assess for changes in the HIV-1 LTR. Biochemical data was used to construct parameters for mathematical modeling to determine relative proportions of the HIV-1 LTR in the various states; repressed (**c**), intermediate (**d**), and activated (**e**) over 120 h. The black line demonstrates the solved value of the original parameter set, while the grey lines are all the realizations with respect to the sampling of parameters using a Latin hypercube sampling method. The dashed green, red and blue lines represent 80%, 90% and 95% confidence intervals, respectively. (**f**) Overlay of all three LTR states; repressed (LTR_*R*_, red), intermediate (LTR_*I*_, blue), activated (LTR_*A*_, green). Selected lanes from the same blot with identical exposure settings are presented in panel a.

We have attempted to mimic three states of the HIV-1 LTR, including a repressed state designated as LTR_*R*_ (repressed), a basal transcription state as LTR_*I*_ (intermediate), and an activated transcriptional state termed LTR_*A*_ (activated) in our mathematical model (Fig. 1; see Materials and Methods section for additional details regarding mathematical model). To resemble LTR_*R*_ in T-cells, infected cells were cultured in low serum media (0.1% FBS) for 36 h, and then placed in 20% FBS media and treated with an inducer (PMA/PHA or IR) to yield a fully activated state (LTR_*A*_). The transcription of cellular and viral genes is initiated by RNAPII, however, shortly after transcription initiation, a regulatory mechanism known as promoter proximal pausing causes RNAPII to stop. Tat, the HIV-1-encoded transcriptional regulator binds the TAR stem loop of the nascent RNA and recruits the p-TEFb complex. The pausing is then alleviated by the assembly of the Tat-TAR-p-TEFb complex on the HIV-1 promoter which allows for synthesis of full-length HIV-1 RNA via CDK9-mediated hyperphosphorylation of RNAPII’s CTD. In addition to this phosphorylation event, p-TEFb’s CDK9 has also been found to phosphorylate Histone H1, an abundant linker histone involved in nucleosome-DNA binding and the maintenance of compact chromatin. Several studies have confirmed the link between Histone H1 phosphorylation and transcriptional activation and suggest that phosphorylation of this protein weakens the binding affinity of Histone H1 to chromatin allowing for it to be removed from transcriptionally active regions^36–39^. Therefore, to measure overall activity and transcription of the cell, an *in vitro* kinase assay was performed using J1.1 whole cell extract using [γ-^32^P]-ATP with Histone H_1_ as a substrate. J1.1 cells are HIV-1 LAI infected Jurkat E6 cells and produce wild-type virus^40^. Results in Fig. 2a show that overall levels of kinase activity in HIV-1 infected T-cells were low at 0 h (Fig. 2a, Lane 1), which was expected due to the presence of low serum media. When T-cells were placed in a 20% FBS media T-cell transcription was activated and active kinase levels increased (6 h, Lane 2). Interestingly, the overall activation nearly returned to basal levels after 24 h (Lane 3). However, when T-cells were activated with an inducer (PMA/PHA or IR), the levels of activation were sustained up to 24 h (Lane 6). Therefore, we reasoned that the transient increase in phosphorylation of Histone H_1_ observed in the presence of 20% FBS media and the absence of an inducer (lanes 1–2) is representative of the occasional transcriptional activation of the HIV-1 LTR to an intermediate state and return to basal transcription (LTR_*R*_; lanes 2–3). Conversely, the more sustained phosphorylation of Histone H_1_ observed in the presence of 20% FBS media and an inducer (lanes 4–5) is representative of the transition from an intermediate state LTR to a fully activated state of the LTR and more rapid return to the intermediate state (LTR_*I*_; lanes 5–6).

We next focused on the forward transitions from LTR_*R*_ to LTR_*I*_ (*k*_*ON*_) and LTR_*I*_ to LTR_*A*_ (*k*_*A*_(Tat)) using a more specific approach to target HIV-1 transcription activation mediated, in part, by CDK9 phosphorylation of Histone H1^41–43^. Here, we immunoprecipitated CDK9/Cyclin T_1_ complexes from infected T-cell whole cell extracts and used samples from 0 and 6 hrs (+/− inducer) to better represent the transitions of the HIV-1 LTR with the absence of an inducer representing the transition to an intermediate state (LTR_*R*_ to LTR_*I*_) and the presence of an inducer representing the transition to an activated state (LTR_*I*_ to LTR_*A*_). We used Histone H_1_ as a substrate as it has previously been shown to serve as a substrate for the p-TEFb complex^36,37^. Results in Fig. 2b show a background level of CDK9 kinase activity at 0 h (lane 1), which may indicate presence of short abortive transcripts, followed by a 34.7% increase at 6 h (lane 2). Similar results were obtained when using a whole cell extract from induced HIV-1 infected cells (20.5%; lane 3). Collectively, these data indicate that the transient transcriptional activation in HIV-1 infected T-cells that produces short HIV-1 RNA transcripts and occasional genomic readthroughs may potentially be a more rapid event allowing the LTR to return to a more quiescent state quickly as compared to the sustained Tat-activated transcription that results in production of infectious virus.

To construct parameter values for the mathematical model (Fig. 1), densitometry counts of the kinase assay (Fig. 2a,b) were taken to determine the rate of transition between each of the HIV-1 LTR states (LTR_*R*_, LTR_*I*_, LTR_*A*_). Briefly, to determine the parameter values *k*_*ON*_ (LTR_*R*_ to LTR_*I*_) and *k*_*A*_Tat (LTR_*I*_ to LTR_*A*_), we utilized densitometry counts of the CDK9-IP kinase assays between 0 and 6 h (Fig. 2b). The transition from LTR_*R*_ to LTR_*I*_ is measured in the absence of an inducer, while the transition from LTR_*I*_ to LTR_*A*_ is measured in the presence of an inducer of viral transcription. Furthermore, the reverse rates (*k*_*OFF*_ and *k*_*I*_; Fig. 1) utilize densitometry counts of the total kinase assay (Fig. 2a) between 6 and 24 h in the absence and presence of an inducer, respectively. Densitometry analysis was normalized as described in Materials and Methods Section.

The results in Fig. 2c demonstrate the proportion of T-cell HIV-1 LTRs which are in a repressed state at a given time over 120 h, as predicted by our mathematical model. The black line demonstrates the value of the original parameter set, while the grey lines are all the realizations with respect to the sampling of parameters using a Latin hypercube sampling method. The dashed green, red and blue lines represent 80%, 90% and 95% confidence intervals, respectively. These results show a sharp decline in the proportion of HIV-1 LTRs in the repressed state following activation, eventually stabilizing at low proportions (1.99%), as expected. Similarly, the results in Fig. 2d show the proportion of T-cell HIV-1 LTRs which are in an intermediate state which results in the basal transcription of HIV-1 RNAs. The LTR_*I*_ state demonstrates unique changes in proportions over time, beginning with 0% of LTRs in an intermediate state followed by a sharp increase with a peak at approximately 21.31 h resulting in 42.96% of the LTRs in an intermediate state. These trends are followed by a decline and subsequent plateau suggesting approximately 5.37% of HIV-1 LTRs are in an intermediate state following activation, which are likely responsible for the persistent transcription of HIV-1 RNAs seen in long-term, cART treated patients^6,32,44^. Interestingly, despite vastly different approaches, these findings are in line with a model described by Razooky *et al*. which encompasses a two-state model of the HIV-1 LTR that incorporates Tat as a feedback mechanism. Both the model presented here and that which is proposed by Razooky and colleagues show a transient state, or a state with low probability, of LTR activation that is independent of Tat^24,45^. The proportion of HIV-1 LTRs in the activated state (LTR_*A*_) is shown in Fig. 2e. As expected, the proportion of LTR_*A*_ steadily increased after activation with 20% FBS media and treatment with an inducer, which resulted in the production of full length, genomic HIV-1 RNA and the production of infectious virions with approximately 92.64% of LTRs in an active state at 120 h. Collectively, the relative proportions of LTR activation states changes over time after induction of the virus in T-cells is presented in Fig. 2f, indicating that there is dynamic activity over a range of time with the most diversity of LTR states occurring between approximately 20–24 h.

### RNA production in T-cells as a function of LTR activation

**Figure 3:**
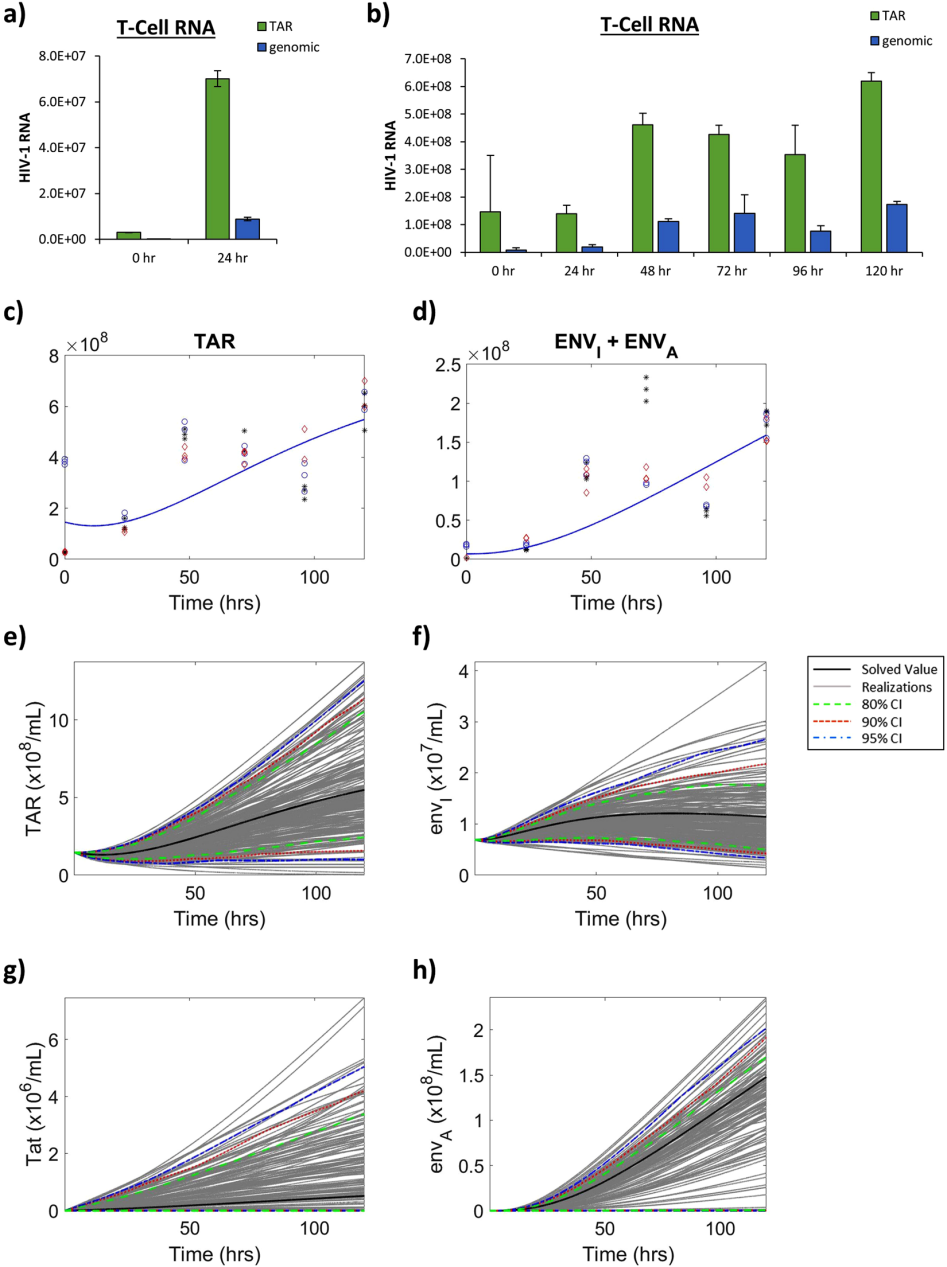
**Viral RNA Production in T-cells**. (**a**) HIV-1 infected T-cells (J1.1) were cultured in 0.1% serum media for 36 h, followed by incubation in 20% FBS media in biological triplicate. At 0 h and 24 h post-induction, RT-qPCR was used to analyze whole cell extracts for TAR RNA and *env* (genomic) RNA. Bars indicate an average of three biological replicates analyzed in technical triplicate ±S.D. (**b**) HIV-1 infected T-cells (J1.1) were cultured in 0.1% serum media for 36 h, followed by incubation in 20% FBS media and treated with an inducer (PMA/PHA) at 0 h in biological triplicate. Samples were analyzed by RT-qPCR in technical triplicate at 0, 24, 48, 72, 96, and 120 h post-induction for TAR and genomic RNA. Values were used to fit parameter rates for TAR (**c**) and *env* (**d**) production from LTR_*A*_. Obtained RT-qPCR values are shown in conjunction with model predictions using solved fitted parameters. Parameter rates for mathematical modeling were constructed using RT-qPCR values and numerical estimations. Constructed parameters were used to mathematically model production of TAR RNA (**e**), *env* RNA from LTR_*I*_ (**f**), Tat protein (**g**), and *env* RNA from LTR_*A*_ (**h**). The black line represents the solved value of the original parameter set, the grey lines are all the realizations with respect to the sampling of parameters using a Latin hypercube sampling method. The dashed green, red and blue lines represent 80%, 90% and 95% confidence intervals, respectively.

We next examined the production of two HIV-1 viral transcripts including TAR RNA, a short, non-coding viral RNA produced as a result of non-processive transcription^46^, and mRNA responsible for the production of the HIV-1 envelope protein, termed *env*, as a marker of full length genomic transcription. As previously described, 3 biological replicates of infected T-cells were cultured in low serum media (0.1% FBS) for 36 h, and then were placed in 20% FBS media. Samples were analyzed at 0 and 24 h following the addition of high serum media by RT-qPCR in technical triplicate for the production of TAR and *env* RNA to determine the production parameters from LTR_*I*_. Figure 3a shows a high level of both TAR and *env* RNA production associated with a repressed transcriptional state (LTR_*R*_, 0 h), with an average of 3.00 × 10^6^ and 8.31 × 10^4^, respectively, from three biological replicates analyzed in technical triplicate. To accurately estimate RNA production from LTR_*A*_, given that the modeled time frame is 120 h, T-cells were cultured as described above with induction using PMA/PHA at time 0. Samples analyzed by RT-qPCR for viral RNA production at 0, 24, 48, 72, 96, and 120 h to determine parameter values for the production of RNA from LTR_*I*_ and LTR_*A*_. Similar to Fig. 3a, the results in Fig. 3b also show a high TAR and *env* RNA associated with a repressed transcriptional state (LTR_*R*_, 0 h), consisting of an average of 1.45 × 10^7^ and 7.08 × 10^6^, respectively, from three biological replicates analyzed in technical triplicate. Taken together, these results suggest that there is an increase in transcription of both short, noncoding RNAs and long genomic RNAs associated with the LTR activation.

To model experimental results mathematically, we determined RNA production rates for each of the defined RNA parameters (Fig. 1). In our model the repressed state of the LTR (LTR_*R*_) does not result in the production of infectious virus as demonstrated by no transcription of full-length genomic RNA (*env*). However, there is persistent, non-processive transcription which results in the production of TAR RNA, as supported by numerous studies which show the presence of TAR RNA in both *in vitro* and *in vivo* samples despite suppressive antiretroviral regimens^6,44,47,48^. These studies have found that intracellular levels during antiretroviral treatment can range from 1 × 10^1^ to 1 × 10^5^ TAR RNA copies *in vivo*^6,44^. Along these lines, we have estimated the production of TAR RNA in infected T-cells to occur at a rate of 2.5 × 10^4^ copies/mL/h (α_*m1R*_). The hourly rates at LTR_*I*_ for TAR RNA production (α_*m1I*_) and *env* RNA production (α_*m2I*_) were constructed using experimentally derived averages at the corresponding time (Table 1). The parameter values for TAR and *env* RNA production from LTR_*A*_, α_*m1A*_ and α_*m2A*_, (Fig. 3c,d) as well as degradation/exit rates of both RNAs were fit to the experimental data in Fig. 3b using the standard least square fitting procedure (see Materials and Methods). The degradation/exit rates can encompass multiple potential mechanisms beyond just degradation. This can include the export of viral RNA out of the cell through such cellular secretory pathways as secretory autophagy and extracellular vesicle release^33,35,44,48,49^. Furthermore, to correlate RNA transcription rates with the expression of HIV-1 Tat protein, we utilized densitometry counts of Tat Western blots (Supp. Fig. 1) at the corresponding times to calculate a Tat production parameter value (α_*p1*_) which is modeled as a function of *env* RNA production from LTRs in the intermediate state. The production of HIV-1 Tat from the repressed and activated LTR state is not included in our model due to predominant production of Tat from early doubly spliced mRNAs which are produced from full length transcription of genomic RNA. Detailed information regarding governing equations is presented in the Materials and Methods section.

**Table 1:** Mathematical Model Parameter Values.

		Calculations	T-Cell	Monocyte/Macrophage
*k*_*ON*_	LTR_*R*_ → LTR_*I*_	[0–6 h Cdk9-IP H1 kinase densitometry counts from Untreated samples]/6	*k*_*ON*_ = 5.785% H1 phosphorylation (densitometry) change/mL/hour	*k*_*ON*_ = 9.245% H1 phosphorylation (densitometry) change/mL/hour
*k*_*OFF*_	LTR_*I*_ → LTR_*R*_	[6–24 h H1 kinase densitometry counts from Untreated samples]/18	*k*_*OFF*_ = 1.220% H1 phosphorylation (densitometry) change/mL/hour	*k*_*OFF*_ = 1.228% H1 phosphorylation (densitometry) change/mL/hour
*k*_*A*_Tat	LTR_*I*_ → LTR_*A*_	[0–6 h Cdk9-IP H1 kinase densitometry counts from Inducer-treated samples]/6	*k*_*A*_Tat = 3.409% H1 phosphorylation (densitometry) change/mL/hour	*k*_*A*_Tat = 9.010% H1 phosphorylation (densitometry) change/mL/hour
*k*_*i*_	LTR_*A*_ → LTR_*I*_	[6–24 h H1 kinase densitometry counts from Inducer-treated samples]/18	*k*_*I*_ = 0.0% H1 phosphorylation (densitometry) change/mL/hour	*k*_*I*_ = 2.451% H1 phosphorylation (densitometry) change/mL/hour
α_*m1R*_	LTR_*R*_ → TAR	[0 h TAR-(−24 h TAR)]/24 ~2.5 × 10^4^ copies/mL/h	α_*m1R*_ = 2.5 × 10^4^ copies/mL/hour	α_*m1R*_ = 2.9 × 10^4^ copies/mL/hour
α_*m1I*_	LTR_*I*_ → TAR	[0–24 h TAR PCR average from Untreated]/24	α_*m1I*_ = 2.8 × 10^6^ copies/mL/hour	α_*m1I*_ = 6.54 × 10^4^ copies/mL/hour
α_*m1A*_	LTR_*A*_ → TAR	Numerically estimated	α_*m1A*_ = 1.37 × 10^7^ copies/mL/hour	α_*m1A*_ = 4.51 × 10^5^ copies/mL/hour
α_*m2I*_	LTR_*I*_ → *env*_*I*_	[0–24 h genomic PCR average from Untreated]/24	α_*m2I*_ = 3.63 × 10^5^ copies/mL/hour	α_*m2I*_ = 8.13 × 10^3^ copies/mL/hour
α_*m2A*_	LTR_*A*_ → *env*_*A*_	Numerically estimated	α_*m2A*_ = 2.47 × 10^6^ copies/mL/hour	α_*m2A*_ = 4.00 × 10^4^ copies/mL/hour
α_*p1*_	*env* → Tat	[(0–24 h Tat densitometry counts from Inducer-treated samples)/24]/3	α_*p1*_ = 0.040% Tat (densitometry) change/mL/hour	α_*p1*_ = 0.038% Tat (densitometry) change/mL/hour
α_*p2*_	*env* → Pr55	[(0–24 h Pr55 densitometry counts from Inducer-treated samples)/24]/3	α_*p2*_ = 0.154% Pr55 (densitometry) change/mL/hour	α_*p2*_ = 0.194% Pr55 (densitometry) change/mL/hour
α_*p3*_	Pr55 → p24	[(0–24 h p24 densitometry counts from Inducer-treated samples)/24]/3	α_*p3*_ = 0.136% p24 (densitometry) change/mL/hour	α_*p3*_ = 0.081% p24 (densitometry) change/mL/hour
γ_*p2*_	p24 → Degradation	0	γ_*p2*_ = 0 p24/mL/hour	γ_*p2*_ = 0 p24/mL/hour
γ_*m1*_	TAR → Degradation/Exit	Numerically estimated	γ_*m1*_ = 1.17 × 10^4^ RNA_1_/mL/hour	γ_*m1*_ = 2.68 × 10^4^ RNA_1_/mL/hour
γ_*m2*_	*env* → Degradation/Exit	Numerically estimated	γ_*m2*_ = 2.24 × 10^3^ RNA_2_/mL/hour	γ_*m2*_ = 5.91x × 10^2^ RNA_2_/mL/hour

According to our mathematical model, the production of TAR RNA is low during the repressed state but upon progression through the intermediate to the active state of the LTR there was sustained, increasing production of TAR RNA (Fig. 3e). The dynamics of *env* (genomic) RNA production from LTR_*I*_ (Fig. 3f) show a slight increase in production starting at approximately 1.49 h and ending at approximately 81.18 h, which correlates to the modeled LTR_*I*_ dynamics. Furthermore, there is a relatively sustained production of approximately 3.63 × 10^5^ copies of genomic RNA from an estimated 5.37% of LTRs that persist in the intermediate state following activation (Fig. 2f). Since the presence of HIV-1 Tat protein is critical for activated transcription, we also investigated the dynamics of Tat expression as a function of time. As the results in Fig. 3g suggest, the production of Tat increases linearly over the modeled time frame measuring 5.19 × 10^5^ proteins at 120 h indicating continual transcription activation resulting in the production of virus. To confirm this, we evaluated the production of genomic RNA (*env*) from a fully activated LTR, which we have modeled to elicit the production of fully infectious virions, over 120 h. The results in Fig. 3g confirm this showing an increase in production of genomic RNA. Following induction there is a steady increase in the number of genomic RNA transcripts with approximately 1.48 × 10^8^ copies at 120 h. Altogether, our model effectively demonstrates the sustained production of small non-coding RNA, regardless of the activation state of the LTR, and the complex dynamics of genomic RNA production as a function of LTR activation.

### LTR activation-dependent protein expression in T-cells

**Figure 4:**
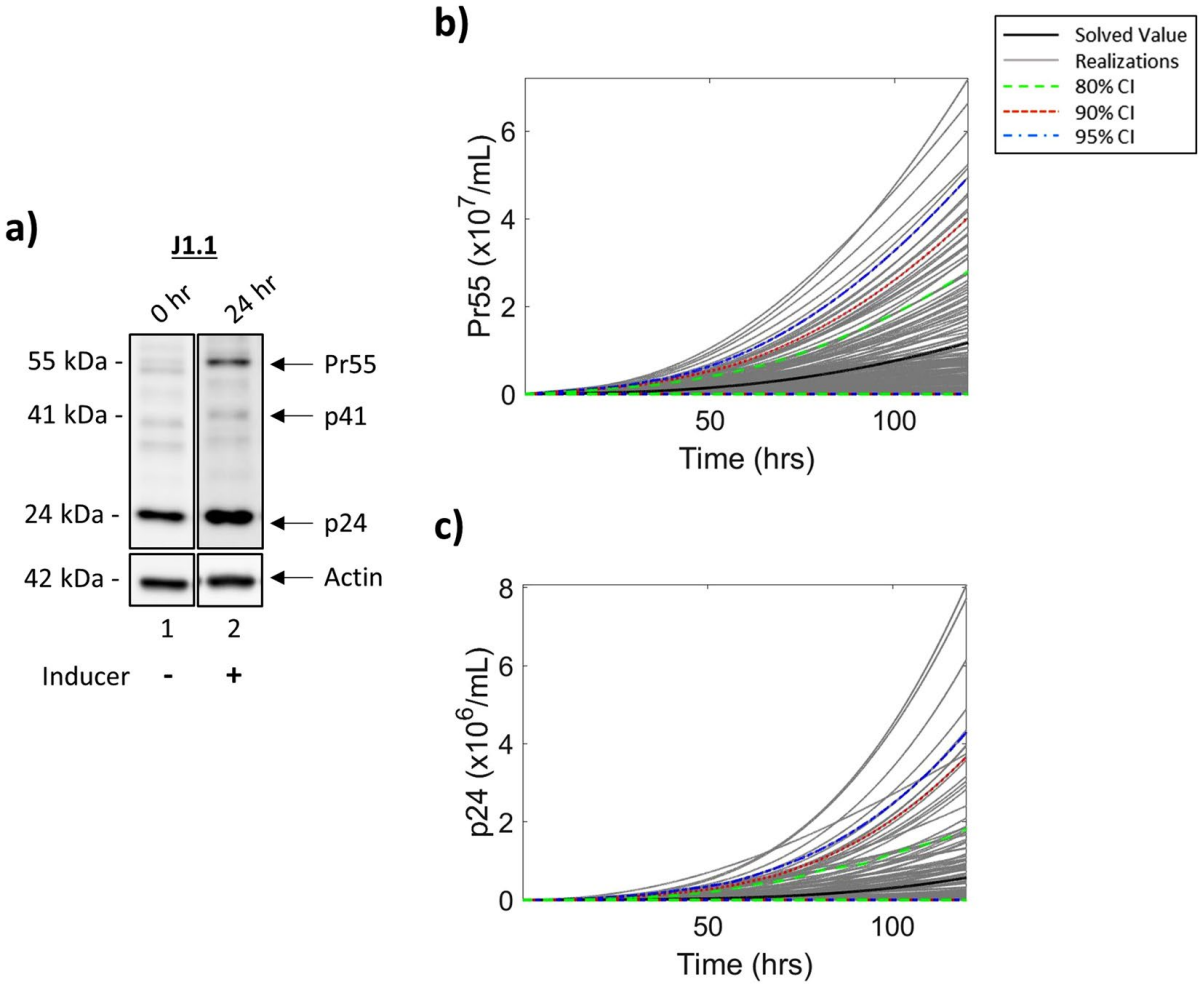
**Viral Protein Production in T-cells**. J1.1 cells were placed in 0.1% FBS media for 36 h, and subsequently placed in 20% FBS media and treated with an inducer (IR). Resulting whole cell extracts were analyzed by Western blot at 0 h and 24 h post-induction for the presence of Pr55 and p24 (**a**). Densitometry counts were normalized and used to determine parameter rates for mathematical modeling of protein production, Pr55 (**b**) and p24 (**c**), over 120 h. The black line indicates the solved value of the original parameter set, while the grey lines are all the realizations with respect to the sampling of parameters using a Latin hypercube sampling method. The dashed green, red and blue lines represent 80%, 90% and 95% confidence intervals, respectively. Selected lanes from the same blot with identical exposure settings are presented in panel a.

Following transcription, the Gag polyprotein (Pr55) is expressed from unspliced genomic RNA. During maturation, Pr55 is cleaved by a virally encoded protease to produce four smaller viral proteins, including the HIV-1 capsid protein, p24, a marker of virus maturation. Along these lines, we examined the production of both Pr55 and p24 in T-cells following incubation in low serum media (0.1% FBS) for 36 h, subsequently cultured in 20% FBS media, and treated with an inducer for 24 h. Whole cell extracts were analyzed by Western blot using a p24 antibody. Not surprisingly, the results in Fig. 4a show a high level of p24 background expression in T-cells at 0 h (lane 1), which was expected due to higher levels of basal transcription in these cells. Furthermore, there is little to no detection of Pr55, suggesting efficient cleavage of the polyprotein to produce p24. Analysis of whole cell extracts at 24 h after introduction of an inducer and the addition of 20% serum media (Fig. 4a; lane 2) indicate an increased production of Pr55 as compared to 0 h samples which subsequently results in the increased production of p24 mediated by viral protease cleavage of the polyprotein.

To determine parameter values for our mathematical model, densitometry counts were taken of the Pr55/p24 Western blot (Fig. 4a) to estimate rates of protein production (α_*p2*_) and subsequent cleavage (α_*p3*_). As it can be assumed that the translation of Pr55 from genomic RNA is unaffected by the introduction of an inducer, α_*p2*_ has equivalent production rate values from both pools of genomic RNA, termed *env*_*I*_ and *env*_*A*_ (Fig. 1). To better represent the system, α_*p3*_, the rate of Pr55 cleavage into p24, has been derived from densitometry counts corresponding to the increase in p24 production over 24 h, rather than the increase in p24 relative to Pr55 production. Additionally, although Fig. 4a demonstrates the presence of p24 at 0 h, we hypothesized that this is unlikely to be production of novel RNAs produced by transcriptional activation coming from LTR_*R*_. To test this, T-cells were cultured in low serum media (0.1% FBS) for 36 h followed by the addition of 20% FBS media. Cells were then lysed using a non-detergent lysis buffer and the lysates and conditioned supernatants were used to treat uninfected T-cells and myeloids +/− an entry enhancer. The results from the infectivity assay in Supp. Fig. 2a show no p24 in recipient cells when treated with lysates or supernatants from transcriptionally latent cells (0 hr; lane 3 and 7, respectively) in the absence an of entry enhancer. Interestingly, when an enhancer is added, p24 is present in recipient cells (lane 4 and 8). However, this p24 is unlikely to represent the production of virus as there is a distinct lack of gp120, the HIV-1 envelope protein. These results suggest that incubation in low serum media effectively induces a transcriptionally repressed state without the production of infectious virus and that the presence of p24 in recipient uninfected cells is likely due to the uptake of Gag-coding mRNA or genomic RNA from the cell lysates either as free RNA or RNA packaged into extracellular vesicles (EVs) ^33,39,48^. Furthermore, these results confirm the production of fully infectious virus at 24 h from LTR_*A*_ (Supp. Fig. 2a, lanes 5–6 and 9–10). These conclusions were confirmed using RT-qPCR (Supp. Fig. 2b–e). For clarity of the proposed model, 0 h p24 values have been set to zero.

The results in Fig. 4b show the modeled dynamics of Pr55 production over 120 h, with no Pr55 associated early time points as 100% of the LTRs are in the repressed state. This is followed by a slow increase in Pr55 production over the modeled time frame resulting in approximately 1.17 × 10^7^ Pr55 at 120 h. Similarly, the expression of p24 shows little fluctuation over the modeled time frame (Fig. 4c). These results suggest there is a slow, but steady increase beginning at 0 h which is sustained throughout 120 h, potentially due to accumulation of Pr55 as the LTR switches from an intermediate state to one of full activation, resulting in 5.67 × 10^5^ p24 at 120 h. Taken together, these results show an increase in the production of both viral proteins, Pr55 and p24, as a result of viral activation.

### Modeling dynamics of LTR activation in macrophages

**Figure 5:**
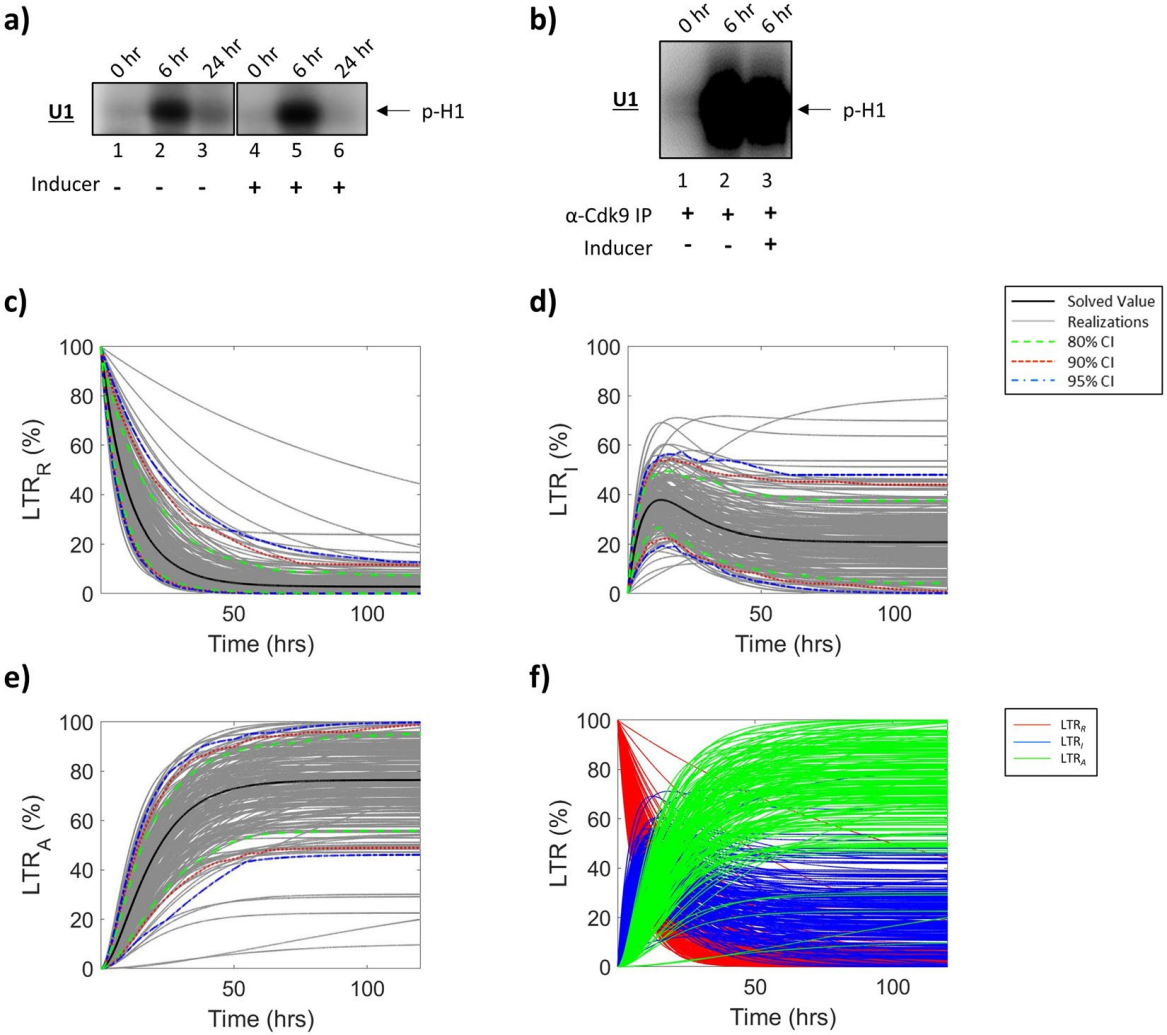
**HIV-1 LTR Dynamics in Macrophages**. U1 (HIV-1 infected myeloids) were placed in low serum media (0.1% FBS) for 36 h, and subsequently incubated in 20% FBS media and treated with an inducer (IR). Whole cell extracts were then analyzed using an *in vitro* total kinase assay (**a**) or a CDK9 IP kinase assay (**b**) to evaluate for changes in the HIV-1 LTR. Biochemical assays were analyzed by densitometry, counts were normalized and used to construct parameters for mathematical modeling to assess relative proportions of the HIV-1 LTR in the various states; repressed (**c**), intermediate (**d**), and activated (**e**) over 120 h. The black line demonstrates the solved value of the original parameter set, grey lines are realizations due to the sampling of parameters using a Latin hypercube sampling method. The dashed green, red and blue lines represent 80%, 90% and 95% confidence intervals, respectively. (**f**) All three LTR states; repressed (LTR_*R*_, red), intermediate (LTR_*I*_, blue), activated (LTR_*A*_, green) are superimposed to indicate changes relative to each state at a given time. Selected lanes from the same blot with identical exposure settings are presented in panel a.

To determine transcriptional differences between HIV-1 infected T-cells and myeloid cells, the same experiment was performed as described previously using U1 whole cell extracts for *in vitro* kinase labeling with [γ-^32^P]-ATP with Histone H_1_ as a substrate. Results in Fig. 5a show that, similar to T-cells, the presence of low serum media elicited low kinase activity levels at 0 h (lanes 1 and 4). When cells were introduced to 20% FBS media there was an observed increase in kinase levels (6 h, lane 2) followed by a reduction in activity at 24 h, although kinase activity levels did not return to basal levels (lane 3) as seen in T-cells (Fig. 2a, lane 3), suggesting sustained transcriptional activation in myeloid cells from the intermediate state of the LTR as compared to T-cells. Upon induction, myeloid cells followed a similar pattern with an increase in kinase activity at 6 h, however, there was a faster return to baseline levels at 24 h in comparison to T-cells (Fig. 5a, lane 6). These results indicate distinct transcriptional timing in myeloid cells as compared to T-cells with myeloid cells having a more sustained level of LTR_*I*_ transcription.

Similar to the T-cell experiments, CDK9/Cyclin T_1_ complex was immunoprecipitated from infected U1 whole cell extracts from 0 and 6 hrs (+/− inducer) and used for an *in vitro* kinase assay. The results in Fig. 5b, show low background levels of CDK9 kinase activity at 0 h, similar to J1.1 (Fig. 2b). Following introduction of 20% FBS media, there was a 55.5% increase in CDK9 activity at 6 h (lane 2) with a similar increase activation (lane 3; 54%) in the presence of an inducer. Taken together, these results demonstrate that basal p-TEFb transcription in HIV-1 infected myeloid cells can be increased upon the introduction of serum and that the activation from basal transcription in myeloid cells may be distinctly different from T-cells, potentially due to differences in transcriptional machinery.

To mathematically model the changes in the HIV-1 LTR dynamics in myeloid cells, parameter values were constructed as previously described. The results in Fig. 5c indicate the proportion of myeloid cell LTRs in a repressed state over the modeled time frame (120 h). These data show that there was in a sharp decline in the relative amount of LTRs in a repressed state following activation of the HIV-1 LTR, resulting in the repression of only 2.77% of LTRs at 120 h. Figure 5d shows the relative proportion of intermediate state LTRs in U1 cells which result in the production of HIV-1 RNAs via basal transcription. Initially following activation with 20% serum, there was a drastic increase in the proportion of LTR_*I*_ which resulted in a peak at 12.43 h post induction yielding approximately 37.80% of HIV-1 LTRs in an intermediate state. The observed increase was followed by a subsequent plateau (20.81%) which was sustained throughout the modeled time frame. Not surprisingly, there was an associated increase in the activated LTR state (LTR_*A*_; Fig. 5e) following induction which resulted in approximately 76.42% of HIV-1 LTRs producing full length, genomic HIV-1 RNA and, in turn, replication-competent virions at 120 h. Altogether, the changes in LTR activation states post-induction in myeloid cells is shown in Fig. 5f. Although the overall patterns of change in LTR states was similar to that of T-cells, these data indicate LTR dynamics unique from that of HIV-1 infected T-cells in terms of timing, relative proportions of LTR states during extended time frames (120 h), and notably less variability (i.e. noise) as indicated by the confidence intervals.

### Production of viral RNA in macrophages

**Figure 6:**
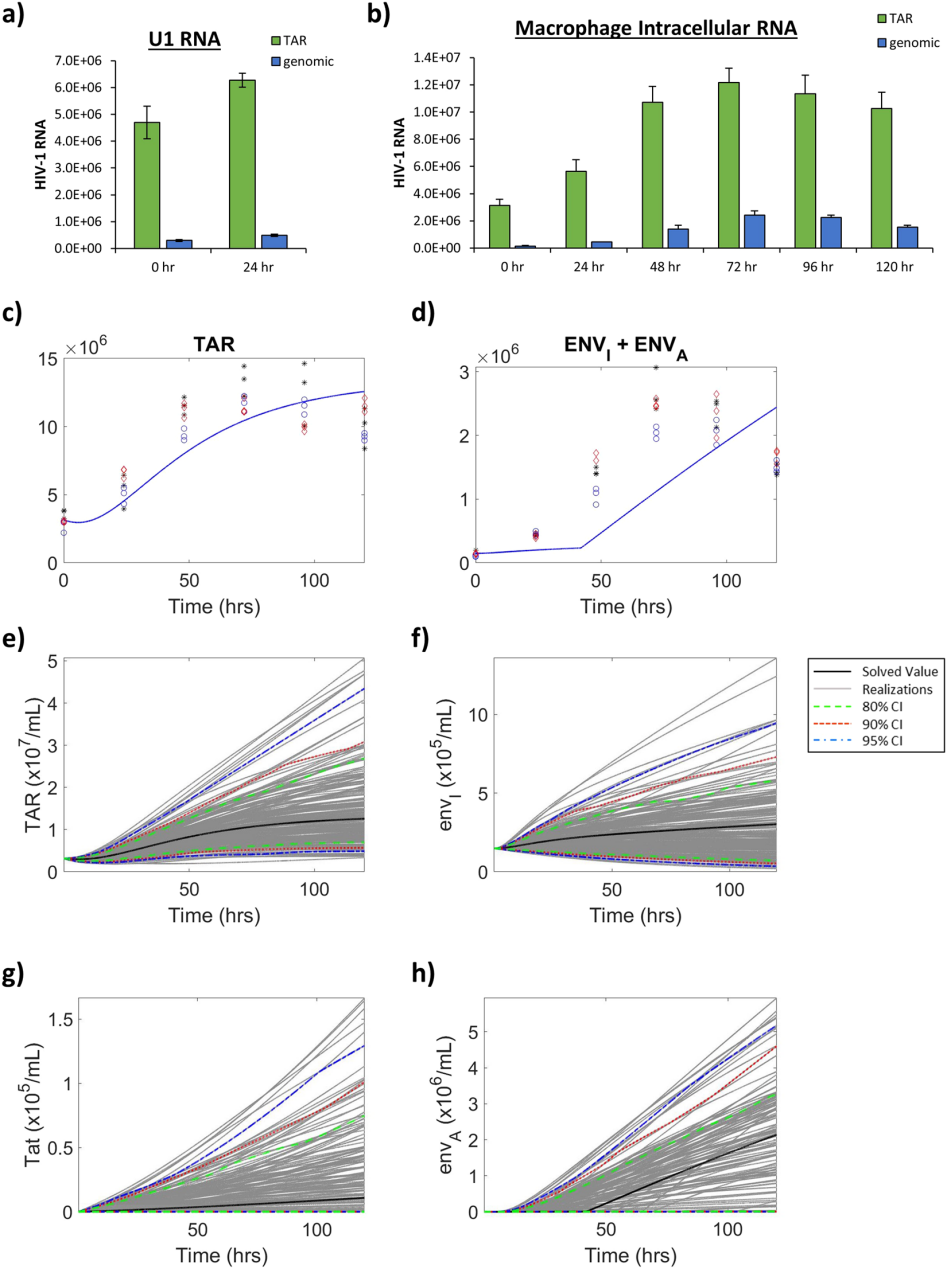
**Production of Viral RNAs in Macrophages**. (**a**) HIV-1 infected myeloids (U1) were cultured in 0.1% serum media for 36 h, followed by incubation in 20% FBS media in biological triplicate. At 0 h and 24 h post-induction, RT-qPCR was used to analyze whole cell extracts for TAR RNA and *env* (genomic) RNA. Bars indicate an average of three technical replicates analyzed in technical triplicate ±S.D. (**b**) HIV-1 infected myeloids (U1) were cultured in 0.1% serum media for 36 h, followed by incubation in 20% FBS media and treated with an inducer (PMA/PHA) at 0 h in biological triplicate. Samples were analyzed by RT-qPCR in technical triplicate at 0, 24, 48, 72, 96, and 120 h post-induction for TAR and genomic RNA. Values were used to fit parameter rates for TAR (**c**) and *env* (**d**) production from LTR_*A*_. Obtained RT-qPCR values are shown in conjunction with model predictions using solved fitted parameters. Parameter rates for mathematical modeling were constructed using RT-qPCR values and numerical estimations. Changes in TAR RNA (**e**), *env* RNA from LTR_*I*_ (**f**), Tat protein (**g**), and *env* RNA from LTR_*A*_ (**h**) were determined using mathematical modeling. The black line represents the solved value of the original parameter set, the grey lines are all the realizations with respect to the sampling of parameters using a Latin hypercube sampling method. The dashed green, red and blue lines represent 80%, 90% and 95% confidence intervals, respectively.

To compare production rates and copy numbers of short, non-coding and full-length HIV-1 viral RNA transcripts, infected U1 cells were cultured as previously described for T-cells. RT-qPCR of three biological replicates analyzed in triplicate was used to examine the production of two HIV-1 viral transcripts, TAR and *env* (i.e. genomic) RNA. For RNA production from LTR_*I*_, samples taken at 0 and 24 h in the absence of an inducer were analyzed and results in Fig. 6a indicate a high level of background transcription of TAR and genomic RNA associated with 0 h (4.70 × 10^6^ and 2.96 × 10^5^, respectively). This was likely due to low levels of basal transcription during a latent state, a finding that is consistent with several *in vivo* studies^6,32,44^. Upon introduction of 20% FBS media, there was little increase in the transcription of TAR (6.27 × 10^6^ copies) and genomic (4.91 × 10^5^ copies) RNAs at 24 h.

RT-qPCR was also performed in technical triplicate to assess for viral RNA production in three biological replicates at 0, 24, 48, 72, 96, 120 h to determine production at LTR_*A*_. Data in Fig. 6b show a high level of background transcription of both RNAs at 0 h (3.13 × 10^6^ and 1.49 × 10^5^, respectively). This was likely due to high levels of RNA prior to transcriptional suppression by low serum media. Upon introduction of 20% FBS media and PMA/PHA, there was an increase in both TAR and *env* RNA in all measured time points relative to 0 h, with a peak in transcripts at the 72 h time point. Collectively, these data show an increase in the transcription of both measured RNAs following introduction of 20% serum media and that myeloid cell transcription is better activated by the introduction of an inducer (PMA/PHA).

To mathematically model our experimental results, we constructed RNA production rates as previously described. Similar to T-cells, we reasoned that the persistent, non-processive transcription observed in several studies involving *in vitro* and *in vivo* analysis of RNA production during antiretroviral therapy suggests that there is TAR RNA production during the repressed state of the HIV-1 LTR^6,44,47,48^. As such, we have estimated the production of short, non-coding TAR RNA in infected myeloid cells to happen at a rate of 2.9 × 10^4^ copies/mL/h (α_*m1R*_). As previously described, rates for TAR (α_*m1I*_) and *env* RNA production (α_*m2I*_) from LTR_*I*_ were determined using experimental data from Fig. 6a. LTR_*A*_ RNA production rates (α_*m1A*_ and α_*m2A*_) as well as RNA degradation rates (γ_*m1*_ and γ_*m2*_) were determined using parameter fitting (Fig. 6c,d). Densitometry counts of Tat Western blots (Supp. Fig. 1) were used to calculate a Tat production value (α_*p1*_).

The results in Fig. 6e demonstrate that the production of TAR RNA occurred in HIV-1 infected myeloid cells regardless of the state of the LTR, as evident by the background level of TAR RNA (3.13 × 10^6^ copies). Furthermore, the production of short, non-coding RNA continued to increase throughout the measured time frame resulting in approximately 1.09 × 10^4^ copies at 120 h. The production of *env* (genomic) RNA from the intermediate state of the LTR (LTR_*I*_) is shown in Fig. 6f. These data indicate a slight increase in the production of full-length genomic RNA from the intermediate state of the LTR over 120 h which yielded an estimated 3.03 × 10^5^ copies from approximately 20.81% of the HIV-1 LTRs which maintained an intermediate state following activation. Similar to T-cells, we have also modeled the production of HIV-1 Tat protein. Figure 6g shows a modest increase in the production of Tat resulting in approximately 1.09 × 10^4^ proteins at 120 h, indicating sustained activated transcription over the modeled time frame and resulting in the increased production of infectious virions. In agreement, Fig. 6h shows an increased production of *env* from an activated LTR state (LTR_*A*_). Initially (0 h), 100% of the HIV-1 LTRs were in a repressed state due to the presence of low serum media. Upon addition of 20% serum media there was a continued increase in the production of *env* which resulted in approximately 2.14 × 10^6^ copies at 120 h. Taken together these results indicate the continuous production of TAR RNA from HIV-1 infected myeloid cells and demonstrates the production of viral RNA as a function of the HIV-1 LTR activation state. Furthermore, these data suggest the magnitude of production of viral products may be inherently different in myeloid lineage cells as compared to T-cells.

### Viral protein production in HIV-1 infected macrophages

**Figure 7:**
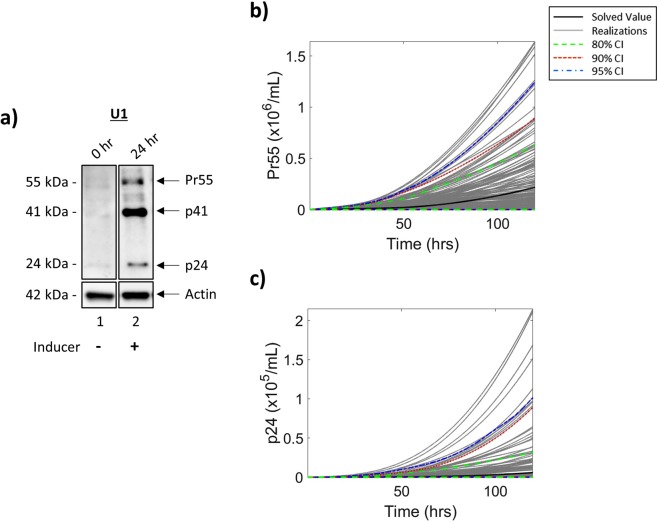
**Production of Viral Proteins in Macrophages**. U1 cells were cultured in serum starved media (0.1% FBS) for 36 h and then placed in 20% FBS media and treated with an inducer (IR). Cells were lysed and whole cell extracts were analyzed by Western blot for the presence of HIV-1 Pr55 and p24 at 0 and 24 h post-induction. Western blots were assessed via densitometry, counts were normalized and used for calculation of protein production parameter values which were utilized for mathematical modeling of viral production of Pr55 (**b**) and p24 (**c**). The black line demonstrates the solved value of the original parameter set, while the grey lines are all the realizations with respect to the sampling of parameters using a Latin hypercube sampling method. The dashed green, red and blue lines represent 80%, 90% and 95% confidence intervals, respectively. Selected lanes from the same blot with identical exposure settings are presented in panel a.

To complement the T-cell experiments, we analyzed the production of Pr55 (Gag polyprotein) and p24 in myeloid cells post-incubation with 0.1% FBS media for 36 h and subsequent treatment with an inducer for 24 h. Following treatment, cells were lysed and analyzed for the presence of these viral proteins using a p24 antibody. The results in Fig. 7a show very low overall levels of Pr55 and p24 background expression in U1 cells at 0 h (lane 1), which is in line with infectivity assay results in Supp. Fig. 2. Interestingly, these levels were markedly reduced as compared to T-cell whole cell extracts at 0 h (Fig. 4a; lane 1). Following treatment with an inducer and the addition of 20% FBS media (Fig. 7a; lane 2) there was an increased production of Pr55 and p24, albeit expression was lower than that of J1.1 (Fig. 4a; lane 2).

To model these biochemical results mathematically, parameter values were constructed as previously described. Figure 7b,c indicate the changes in viral protein production over 120 h including the presence of no Pr55 (Fig. 7b) and p24 (Fig. 7c) at 0 h due to 100% of the HIV-1 LTRs in the repressed state (LTR_*R*_). The data in Fig. 7b show a slow, yet steady, increase in the production of Pr55 following activation of the LTR by 20% serum and introduction of an inducer, which resulted in approximately 2.17 × 10^5^ Pr55 at 120 h. The same trend was observed for p24 levels which yielded an estimated 5.84 × 10^3^ p24 proteins (Fig. 7c). Not surprisingly, the data suggests overall lower levels of Pr55 and p24 in myeloid cells as compared to T-cells at extended time points. Interestingly, the 2-log difference between cell types is maintained for both Pr55 and p24 suggesting similar rates of Pr55 polyprotein cleavage in both cell types.

### Comparing transcriptional differences in HIV-1 infected T-cells and macrophages

**Figure 8:**
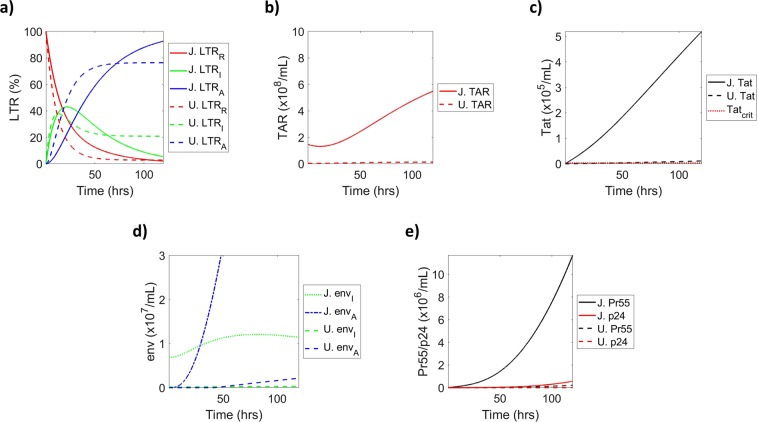
**Comparison of T-cell and Macrophage** **Transcription Activation and Viral Product Production**. Solved values using the original parameter set for J1.1 and U1 are superimposed to illustrate differences in the changes of relative proportions of LTRs over time (**a**), as well as differences in the magnitudes of TAR RNA production (**b**), Tat protein levels (**c**), *env* RNA production (**d**), and Pr55/p24 levels (**e**). Solid lines indicate T-cell values and dashed lines indicate myeloid values over 120 h.

To address differences in LTR state dynamics amongst cell types, solved parameter values for myeloid and T-cells were superimposed to determine timing and magnitude differences between cell types. The data in Fig. 8a demonstrates that myeloid LTRs exhibit faster changes in LTR states as indicated by rapid exit of the LTR from the repressed state, complemented by quick entry into an activated LTR as compared to T-cells. These cell-type differences are supported by a 2018 study that utilized a multiply spliced transcript reporter assay to measure the generation of RNA over time and found that primary myeloid cell multiply spliced transcripts, such as those which would be generated by LTR_*I*_, peaked more quickly than T-cells^50^. This trend was validated in primary latent peripheral blood mononuclear cells (PBMCs), which were separated into T-cell and myeloid populations and measured for rates of LTR state activation by the same methods as previously described for our cell lines (Supp. Fig. 3). Furthermore, these data show a more heterogeneous population of LTR states in myeloids given that, at extended time frames (120 h), approximately 76.42% of LTRs were in the activated state (dotted blue line), 20.81% of LTRs were in an intermediate state (dotted green line), with the remaining 2.77% in a repressed state (dotted red line) following activation. Conversely, T-cells exhibited 92.64% of LTRs in an activated state (blue line) post-induction, with only 5.37% and 1.99% in intermediate (green line) and repressed (red line) states, respectively. Not surprisingly, the production of short, non-coding RNA (TAR) was increased in T-cells (solid red line) as compared to myeloid cells (dotted red line; Fig. 8b), likely due to the larger proportion of T-cell LTRs in the activated state. Tat production, produced from LTR_*I*_ in our model, closely follows a similar trend (Fig. 8c), due to a lower calculated production rate for myeloids despite a larger proportion of myeloid LTRs in an intermediate state at 120 h. The production of full-length genomic RNA from both LTR_*I*_ (green) and LTR_*A*_ (blue) in T-cells and myeloid cells is shown in Fig. 8d. As expected, the production of *env*_*I*_ (green lines) was higher than *env*_*A*_ (blue lines) in both T-cells and myeloids at early time points. Interestingly, the transition from a predominately *env*_*I*_ state to a predominately *env*_*A*_ state occurred earlier in T-cells (approximately 28.5 h) than myeloid cells (approximately 50 h) despite the more rapid change in LTR state exhibited by myeloid cells. This is likely due to the high rate of RNA production in T-cells as compared to myeloids. Finally, the production of HIV-1 viral proteins, Pr55 and p24, was increased in T- cells as compared to myeloid cells (Fig. 8e). Collectively, these results suggest that T-cells produce viral products at higher levels compared to their myeloid counterparts although the myeloids show a more rapid change in the LTR state.

### Modeling the use of F07#13

**Figure 9:**
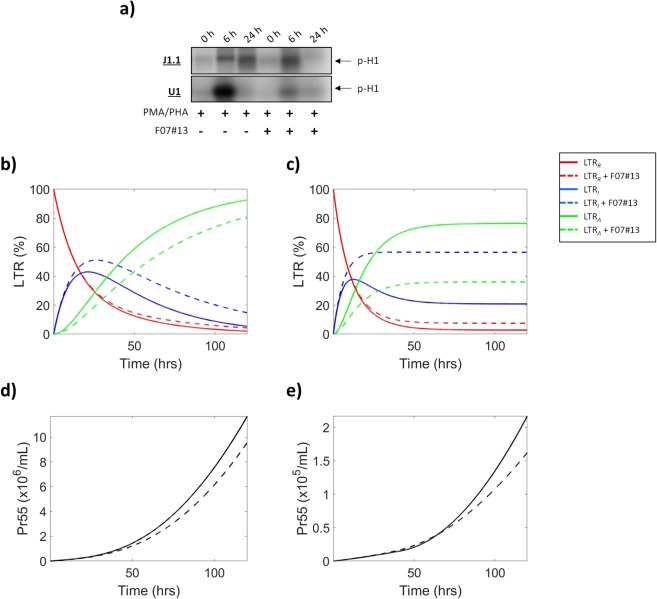
**Introduction of F07#13 Induces Changes in HIV-1 LTR Dynamics**. J1.1 (HIV-1 infected T-cells) and U1 (HIV-1 infected myeloid) cells were placed in a repressed state using low serum media for 36 h and subsequently induced using 20% serum media and PMA/PHA. At the same time, cells were treated with F07#13. An *in vitro* kinase assay with Histone H_1_ as the substrate was used to analyze overall transcriptional activity of the cell at 0, 6, and 24 h post-induction (**a**). Densitometry was used to analyzed relative changes in LTR activation and resulting counts were normalized and used to construct parameter values. Mathematically-solved predictions from the three-stage model of LTR activation in J1.1 (**b**) and U1 (**c**) cells shows the relative proportions of three LTR states [repressed (LTR_*R*_; red line), intermediate/basal transcriptional state (LTR_*I*_; blue line) and activated (LTR_*A*_; green line)] over a 120 h simulation. The graph depicts F07#13 induced changes in LTR dynamics [LTR_*R*_ (dotted red line), LTR_*I*_ (dotted blue line), and LTR_*A*_ (dotted green line)] over the same time frame. Modeling predictions for Pr55 for T-cells (**d**) and monocytes (**e**) are also shown. Solid lines represent model predictions in the absence of F07#13 and dotted lines represent model prediction sin the presence of F07#13.

The dependence of activated viral transcription on the HIV-1 Tat protein makes it an attractive target for therapeutics aimed at inhibiting transcription of the HIV-1 virus. As a result, Tat peptide mimetics, specifically F07#13, have been developed and shown to inhibit transcription both *in vitro* and *in vivo*^15,51^. To further test our model, we tested transcriptional changes associated with the introduction of the transcriptional inhibitor F07#13. F07#13 treatment was incorporated into the governing equations of the model within the terms *k*_*A*_(Tat) and *k*_*I*_ (Fig. 1)_._ Data in Fig. 9a show low levels of kinase activity at 0 h (Lanes 1 and 4), indicating a repressed transcriptional state of the HIV-1 LTR. When cells were induced (20% FBS media/PMA/PHA) the addition of F07#13 drastically mitigated the increase in transcriptional activity observed in HIV-1 infected T-cells in the absence of F07#13 at 24 h (J1.1; lanes 3 and 6). Interestingly, F07#13 treatment elicited a reduction in p-Histone H1 (i.e. maximal drug effect) in HIV-1 infected myeloids at 6 h post-induction (U1; lanes 2 and 5), suggesting differences in transcription between cell types and variation in drug efficacy. The simulation data in Fig. 9b,c show the mathematically derived prediction of the relative proportions of LTR states ± F07#13 in T-cells and myeloids, respectively. As described in Figs. 2f and 5f, in the absence of F07#13, there was a rapid decrease in the number of LTR_*R*_ (red line) concomitant with a sharp increase in the proportion of LTR_*I*_ (blue line) until ~21.31 h and 12.43 h in T-cells and myeloids, respectively. Furthermore, there was an associated increase in activated LTRs (green line) following induction. The graphs show a decrease in the proportional of LTRs in the active state in response to F07#13 treatment (dotted green line). Interestingly, there was a slight increase (9.35%) at 120 h in the relative amount of LTRs in the basal transcriptional state (LTR_*I*_; dotted blue line) observed in T-cells (Fig. 9b) which is evident at approximately 9 h and was sustained through the modeled time frame. In myeloid cells there was a larger increase (35.66%) at 120 h in the relative proportion of LTR_*I*_, evident at approximately 6 h, which was similarly sustained throughout the 120 h time frame. Finally, there was a slight increase in the number of LTRs in latency (LTR_*R*_; dotted red line) in both cell lines, suggesting preferential basal transcription during F07#13 treatment and thereby the production of non-coding RNAs such as TAR. In agreement with our biochemical assays, graphs generated from our model demonstrate the F07#13-mediated LTR effect in T-cells peaks at ~24 h (Fig. 9a) whereas the same effect was observed earlier in myeloid cells (6 h; Fig. 9a). To illustrate the potential utility of the model to explore the effects of F07#13 on the production of viral proteins and, in turn, infectious virions, the production of Pr55 in the presence of F07#13 was modeled and compared to the production of the protein in the absence of the drug. These data show the post-treatment outcomes for T-cells (Fig. 9d) and myeloid cells (Fig. 9e) in terms of viral protein production. Interestingly, these results indicate that F07#13 is more effective in lowering virus production in myeloid cells as compared to T-cells despite a decrease in the relative proportion of LTR_*A*_ in both cell types. The decrease efficacy in T-cells is likely due to compensation by LTR_*I*_ in the production of *env* resulting in no change in the production of Pr55. These data are in line with our previously published results which found that RNAPII was still present on the HIV-1 promoter post-treatment with F07#13 using an *in vitro* transcription assay^31^. Conversely, the addition of F07#13 in myeloid also decreases the relative proportion of LTR_*A*_, however, unlike T-cells this proportionate drop outweighs the relative increase in the proportion of LTR_*I*_.

## Discussion

**Figure 10:**
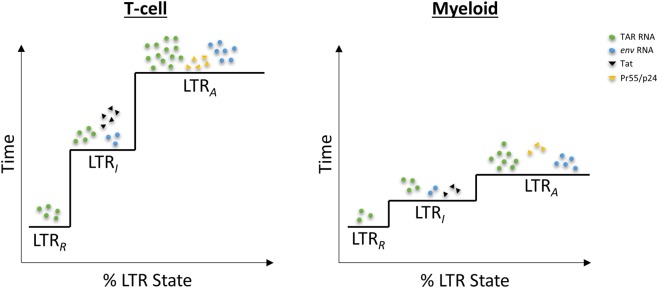
**Summary Model**. The three LTR states for T-cells and myeloid cells are illustrated as a percentage over time. The relative proportion of viral products are depicted as indicated in the key over the relevant LTR state where each is mostly produced. TAR RNA (green circle) is produced at all three states, *env* RNA (blue circle) is produced at LTR_*I*_ and LTR_*A*_, Tat (black triangle) is produced only at LTR_*I*_, and Pr55/p24 (yellow triangle) are produced only at LTR_*A*_. Furthermore, the height of each step to a new LTR state signifies the approximate amount of time required to progress to the next stage of activation. Finally, the width of each LTR “step” indicates the relative percent of the LTR state post-activation.

Here, we have generated a mathematical model to describe three states of HIV-1 transcription. This model was created in such a way that it is open to broad applications. We utilized 100% HIV-1 infected cell lines of two different immune cell types to show differences in the production magnitude and induction speeds between infected T-cells and myeloids. Specifically, our model demonstrates that latent infected myeloids become activated sooner than T-cells under the influence of a transcriptional inducer; however, latent infected T-cells generate more viral products than myeloids, despite a slower activation rate (Fig. 10). Interestingly, the rates of LTR activation in primary cells followed a similar trend to that seen with cell lines, although the overall rates were lower (Supp. Fig. 3). This could potentially be due to the fact that the primary cells were infected *in vitro* and likely not 100% infected. The fact that myeloid and T-cells show variances in the speed of LTR state change and levels of viral components produced points to differences in transcriptional machinery between the cell types. This notion has been supported by previous findings that have shown many differences in transcriptional machinery components, particularly host transcription factors, between myeloid and T-cells, which has been previously described at length by Rohr *et al*.^52^. The lack of an FDA-approved transcription inhibitor for HIV-1 illustrates the need for further research into compounds with the potential to limit viral transcription. As a result, we have integrated potential therapeutic interventions, such as F07#13, into our model to explain their effects on HIV-1 activation and generation of viral products.

The observed high production of TAR RNA in both cell lines (10^6^ to 10^8^) at all time frames as compared to the relative levels of other viral proteins and RNAs, suggests that regardless of cell type, HIV-1 produces short, non-coding RNAs (Figs. 3e and 6e) from all states of the LTR. These findings are confirmed by several patient and *in vitro* studies which show the presence of TAR RNA within infected cells despite antiretroviral therapy, which is effective in reducing other viral products and suppressing the virus in the plasma^6,32,44,48^. Several studies have previously shown that TAR RNA is released from HIV-1 infected cells, including during latent infection, which can then induce numerous inflammatory responses in neighboring cells^33,35,48^.

Other research suggests that during activated transcription, which results in the production of genomic RNAs, the ratio of TAR:Tat:Cyclin T_1_ is 1:1:1^53–55^. Furthermore, the presence of Cyclin T_1_ greatly enhances the binding affinity of Tat to TAR, with a Hill coefficient of 2.7 and a dissociation constant (*K*_*D*_) of 2.45 nM^56^. However, we propose that the production of TAR RNA as a short non-coding RNA within the cell could result in the binding of TAR RNA to Tat protein in the absence of Cyclin T_1_, potentially resulting in TAR RNA produced at high copy numbers to inhibit Tat-activated transcription. Along these lines, our experimental data show a relative abundance of TAR RNA in infected cells as compared to other viral products (i.e. Tat), which could potentially indicate that TAR RNA could be utilized to sequester Tat protein, thereby slowing activated viral transcription and promoting latency of the virus. Altogether, this finding may potentially point to a novel mechanism of viral maintenance of latency.

It has been previously shown that TAR RNA is released in EVs such as exosomes both *in vitro* and *in vivo* even under antiretroviral regimens^33,35,44,48^. Therefore, the γ_*m1*_ and γ_*m2*_ parameters include exit of viral products from the cell through these secretory mechanisms. Additionally, the fact that there is a high levels of TAR RNA in T-cell EVs may suggest enhanced efficiency of T-cells in the packaging of EV products. The data in Supp. Fig. 5 support this hypothesis and demonstrate that the EV-associated viral RNA copy number (white bars; TAR, Supp. Fig. 5a; *env*, Supp. Fig. 5b) is higher in T-cells as compared to myeloids. Furthermore, there are innate differences in the distribution of viral RNA (TAR and *env*) within T-cells and myeloids. T-cells exhibit approximately equal copy numbers of both TAR (Supp. Fig. 5a) and *env* (Supp. Fig. 5b) within the nucleus (black bars) and cytoplasmic fractions (grey bars) while myeloids show a higher proportion of TAR (Supp. Fig. 5a) and *env* (Supp. Fig. 5b) in the cytoplasmic fraction in comparison to the nuclear fraction. These data may suggest differences in the nuclear export of viral RNA which could potentially lead to variations in protein expression as previously demonstrated by the model. Additionally, these data imply that a TAR-mediated Tat inhibition could potentially play a larger role in T-cell latency as compared to myeloids. These observations reiterate broad utility of the model shown herein such as the far-reaching effects of viral transcription in various cell types and potential impacts on viral pathogenesis.

An interesting finding within our biochemical assays showed that there were background levels of p24 consistently produced from latent T-cells suggesting persistent production of mature Gag. Previously, we have shown that exosomes contribute to the lack of transcriptional latency within HIV-1 infected cells^32^. Therefore, the presence of p24 despite a serum-starvation induced latent state further supports the notion that there may not be a true transcriptional latency during HIV-1 infection of T-cells (Fig. 4a). This is in contrast with our observation in myeloid cells (Fig. 7a) which showed minimal levels of p24 in serum-starved cells suggesting different mechanisms of maintaining viral latency between these cell types. This could pose a potential hurdle in designing targeted antiretrovirals and the development of future transcription inhibitors against HIV-1.

The mathematical model presented in this paper is novel in that it incorporates new biological mechanisms in a way that preserves important biologically realistic features. Work is currently under way to study analytical properties of this system, and several important facts have been already established. For example, starting with a positive amount of LTR_*R*_, positivity of all variables is maintained during evolution. Furthermore, all variables are bounded, which is consistent with the fact that no realistic environment can support indefinite exponential growth. During the construction of the model, some simplifying assumptions were made regarding the switching function, 𝑘_*A*_(Tat) and 𝑓_*𝑚2*_(Tat), and the production/decay parameter. This is reasonable because, for a short time frame (5 d), step-wise constant rate approximation is sufficient for capturing the complexity of the system behavior. However, we speculate that nonlinear forms of the switching functions and more biologically realistic forms of the rates of change would be more suitable to study the long-term behavior of the system. While simplistic, the linear system allows for a more thorough understanding of the transient modes, for instance, it may be helpful in clarifying how different rates contribute to the dynamics of each variable at the beginning of transcription. In this manuscript, parameter estimations were carried out for a relatively small subset of parameters that cannot be directly inferred from the data. In future work, a more thorough sensitivity analysis can be performed to establish the role of each of the parameters.

In the future, additional cell types, including primary cells and other immune cell types, or various drug treatments designed to act at multiple stages of latent virus reactivation may be easily integrated into the model we present here to show their downstream effects and optimal time frame for usage. To demonstrate this utility, we have integrated the transcription inhibitor F07#13 into the model to show elicited changes in LTR activation post treatment, which varied between cell types. Specifically, F07#13-repressed transcription was more quickly achieved in myeloid cells in comparison to T-cells (Fig. 9b,c). Furthermore, the differences in the relative proportions of LTRs during various time points may dictate the efficacy of the drug in terms of virus production (Fig. 9d,e). One of the potential applications of the proposed model is the study of the effectiveness of drugs in silico. While this type of work can be carried out numerically on a case-by-case basis, we suggest that valuable insight may be derived by first studying the model analytically and establishing conditions when a certain drug is effective, especially when it is used in combination with other drugs. Other applications of the dynamics of this system or its nonlinear version may potentially be extended to other retroviruses including endogenous ones that are known to activate under given stimuli. An example of this could be the activation of human endogenous retrovirus type-K (HERV-K) by HIV-1 Tat^57^. Moreover, the model could be used to more closely study the various states of the HIV-1 LTR (repressed, intermediate, and activated). For the repressed state of the LTR, these studies could include exploring the effects of various suppressive transcription factors, transcriptional gene silencing mechanisms, and suppressive chromatin complexes on relative proportions of the LTR transcriptional state and the production of viral RNAs and proteins. Inquiries into the intermediate state of the LTR such as RNAPII phosphorylation, basal transcription factors, chromatin looping, and enhancers which could also be incorporated into the model. Additionally, Tat-activated transcription studies such as RNAPII elongation through various genome regions and RNA processing (capping, splicing, 3′end processing) could also be explored. For example, Yukl *et al*. has designed a set of primer and probe sequences which could be easily incorporated to increase the resolution of the model presented here by analyzing several different RNA transcripts. This approach could aid in fine-tuning the model as it includes the use of droplet digital PCR to measure transcripts associated with various blocks in transcription which would allow for more precise inclusion of drug mechanisms^58^. Finally, the presented model could be used to larger data sets regarding integration sites of the HIV-1 genome or other viral genomes and the downstream effects in terms of transcriptional regulation of viral products using chemical or other biological compounds that serve as therapeutics.

## Materials and Methods

### Cell culture and reagents

U1 (HIV-1 infected promonocytic cell with 2 copies of virus; one wild-type and one mutant) and J1 (HIV-1 infected T-cell with one copy of wild-type virus) cells were cultured and maintained in RPMI 1640 supplemented with 10% fetal bovine serum (FBS), 1% penicillin/streptomycin, and 1% L-glutamine (Quality Biological) and incubated in 5% CO_2_ at 37 °C. U1 cells were activated with phorbol 12-myristate 13-acetate (PMA; 1 µM) and phytohemagglutinin (PHA; 10 µg/µL) and then treated with F07#13, an allosteric Tat/CDK9 inhibitor (1 µM), or irradiation (5 Gy). Treated cells were incubated in 5% CO_2_ at 37 °C. Both cell lines (U1 and J1) were provided by the AIDS Reagent Program (National Institutes of Health). Both U1 and J1 cells were serum starved for 3 days, washed, counted, and plated in 20% FBS media +/− inducer. Samples were collected at 0, 6, 12, 24, 48, 72, 96, and 120 h post-induction. The 0 h sample represents samples that were collected 10 minutes post-treatment due to the size of experimental design and handling procedures. Therefore, these samples would have shown some level of transcription within the first 10 min, since the rate of RNA Pol II transcription is fairly rapid and high (the RNAPII complex moves through very long introns and also through regions dense with alternating exons and introns at an average rate of ∼3 kb per min)^59^. Peripheral blood mononuclear cells (PBMCs) were treated with PHA/PMA and IL-2 treatment. After one week, they were infected with HIV-1 (89.6; multiplicity of infection = 3.0). Adherent macrophage cells were separated from T-cells using centrifugation, and 3 days post-infection, T-cells were treated with cART and IL-7, which allowed cells to enter latency. Adherent macrophages were also treated with cART to obtain latent cells. Prior to activation with 20% FBS media and PMA/PHA, cells were kept in low serum media to suppress basal transcription.

### Kinase assay

*In vitro* kinase assays were carried out using whole cell extract (prepared as described below). Total lysates and immunoprecipitated samples were washed three times with appropriate TNE buffer (10 mM Tris, 100 mM NaCl, 1 mM EDTA) and kinase buffer. The reaction mixtures (approximately 30 μl) contained the following final concentrations: 40 mM β-glycerophosphate (pH 7.4), 5% glycerol, 7.5 mM MgCl^2^, 1 mM orthovanadate, 7.5 mM EGTA, [γ-^32^P]-ATP (0.4 mM, 1 μCi), 50 mM NaF, and 0.1% (v/v) β-mercaptoethanol. Phosphorylation reactions were performed with total lysates and immunoprecipitated material and [γ-^32^P]-labeled histone H1 (0.5 μg) as a substrate in threonine tyrosine kinase buffer containing 50 mM HEPES (pH 7.9), 10 mM MgCl2, 6 mM EGTA, and 2.5 mM dithiothreitol. Reactions were incubated at 30 °C for 1 h, stopped by the addition of 1 volume of Laemmli sample buffer (5% β-mercaptoethanol), and run on 4–20% SDS-polyacrylamide gel. Gels were subjected to autoradiography followed by quantification using PhosphorImager software (Amersham Biosciences).

### Infectivity assay

HIV-1 infected cells (1 × 10^6^) J1.1 sample were cultured and then starved in supplemented RPMI media containing 0.1% FBS for 3 days. Cells were centrifuged (325 × g) for 5 min and resuspended in supplemented RPMI media containing 20% FBS for 0 and 24 h incubations periods. Cells were obtained and spun down for 5 min, washed with PBS, followed by cell lysis using 100 µL non-detergenic lysis buffer (50 mM ammonium bicarbonate (pH = 7.8)), Pierce™ Protease and Phosphatase Inhibitor Mini Tablets (1 tablet per 10 mL lysis buffer), and 100 mM NaCl solution. Cellular debris was pelleted and discarded. A total of 1/5th of the intracellular lysate material was treated (±5 µL) Infectin^TM^ (Virongy, LLC, Manassas, VA) onto uninfected Jurkat T cells (1 × 10^6^), uninfected CEM T cells, and uninfected U937 monocytes (data not shown) for a total of 3 days. Cells were pelleted and supernatant was nanotrapped using NT80/82/86 beads (to collect viral EVs and viruses) for Western blot and RT-qPCR analysis.

### RNA isolation, generation of cDNA, and quantitative real-time PCR

For quantitative analysis of HIV-1 RNA, total RNA was purified from infected cell pellets in triplicate using Trizol Reagent (Invitrogen) according to the manufacturer’s protocol. Total RNA was used to generate cDNA with the GoScript Reverse Transcription System (Promega) using specific reverse primers, Envelope Reverse: (5′-TGG GAT AAG GGT CTG AAA CG-3′; Tm = 58 °C) and TAR Reverse: (5′- CAA CAG ACG GGC ACA CAC TAC -3′, Tm = 58 °C). Quantitative real-time PCR analysis was performed with 2 μl of undiluted aliquots of cDNA using iQ supermix (Bio-Rad) with the following pair of primers specific for target TAR sequences: TAR- Reverse: (5′- CAA CAG ACG GGC ACA CAC TAC -3′, Tm = 58 °C) and TAR-Forward (5′- GGT CTC TCT GGT TAG ACC AGA TCT G -3′, Tm = 60 °C). Serial dilutions of DNA from 8E5 cells (CEM T-cell line containing a single copy of HIV-1 LAV provirus per cell) were used as the quantitative standards. The PCR conditions were as follows: one cycle at 95 °C for 2 min, 41 cycles at 95 °C for 15 s and 58 °C for 40 s. Real-time PCR reactions were carried out in triplicate using the BioRad CFX96 Real Time System. Quantification of the samples was determined based on the cycle threshold (Ct) value relative to the standard curve.

### Preparation of whole cell extracts and western blot analysis

Infected cell pellets were harvested and washed with 1X PBS without calcium and magnesium. The resulting pellet was resuspended in lysis buffer [50 mM Tris-HCl (pH 7.5), 120 mM NaCl, 5 mM EDTA, 0.5% Nonidet P-40, 50 mM NaF, 0.2 mM Na_3_VO_4_, 1 mM DTT, and 1 complete protease inhibitor mixture table/50 mL (Roche Applied Science, Mannheim, Germany)]. The mixture was incubated on ice for 20 min with vortexing every 5 min followed by separation via centrifugation at 10,000 × g at 4 °C for 10 min. Lysate total protein concentration was assessed using Bradford protein assay (Bio-Rad).

For analysis, sample lysates (10–20 µg) were added to SDS sample buffer supplemented with 10% 2-mercapthoethanol, heated at 95 °C for 3 min, vortexed, and spun (15,000 × g) for 10 sec until fully collected. Approximately 10 µl of each sample was loaded onto a 4–20% Tris/glycine gel (Invitrogen), run at 200 V, and transferred onto Immobilon PVDF membranes (Millipore) at 0.05 Amp overnight. Membranes were then blocked in 5% milk in PBS with 0.1% Tween-20 (PBS-T) for 2 hours at 4 °C, then incubated overnight at 4 °C PBS-T with the appropriate primary antibody α-p24 (Cat# 4121; NIH AIDS Reagent Program), α-Tat (Cat# 705; NIH AIDS Reagent Program), α-Actin (ab-49900). Membranes were incubated with the appropriate HRP-conjugated secondary antibody for 2 h at 4 °C. HRP luminescence was activated with Clarity Western ECL Substrate (Bio-Rad) and visualized by the Molecular Imager ChemiDoc Touch system (Bio-Rad). Images were quantified using densitometry (ImageJ).

### Isolation of nuclear and cytoplasmic compartments

Cytoplasmic and nuclear compartments were separated and extracted using the NE-PER Nuclear and Cytoplasmic Extraction Reagent kit (Thermo Fisher Scientific) as per the manufacturer’s instructions. Briefly, log-phase J1.1 and U1 cells were collected and resulting culture supernatant was used for exosome isolation as described below. Cell pellets were washed in 1X PBS and incubated with 100 µL of cold CER I followed by vortexing (10 seconds) and subsequent incubation on ice for 10 min. Following incubation, 5.5 µL ice cold CER II was added to both samples and vortexed for 10 seconds followed by a 1 min incubation on ice. Samples were then vortexed, centrifuged for 5 min at 16,000 × g to separate the cytoplasmic compartment which was transferred to a new, pre-chilled tube. Pelleted nuclei were treated with 50 µL of ice cold NER, vortexed for 20 seconds and incubated on ice for 45 min with additional vortexing (every 10 min). Following incubation, samples were centrifuged at 16,000 × g for 10 min and resulting nuclear extract was transferred to a new prechilled tube. Both cytoplasmic and nuclear fractions were then subjected to subsequent RNA isolation, reverse transcription, and qPCR as described above.

### Isolation of exosomes

J1 and U1 culture (five days post last feed; late log phase of growth) supernatant was harvested and enriched for exosomes using Nanotrap particles (NT80/82) as previously described^32–35,48,60^. Briefly, 30 µL of a 30% slurry of NT80/82 (1:1) was added to 1 mL of cell culture supernatant and incubated with rotation overnight at 4 °C. The next day NTs were pelleted and washed with 1 mL of sterile 1X PBS and subjected to RNA isolation, reverse transcription, and RT-qPCR as described above.

### Densitometry

Raw densitometry counts were obtained using ImageJ software. Exposures were matched between membranes according to positive control signals. Densitometry data was normalized by a two-step process. First, the background measurements for each membrane were subtracted from each band of interest. Second, each protein band’s raw densitometry count was converted to a percentage by dividing it by the background measurement for each membrane and multiplying by 100%. This normalization accounted for differences in the backgrounds and exposures between membranes, while also converting densitometry counts to an appropriate unit for use in our model.

### Statistical analysis

Standard Deviations (S.D.) were calculated from technical triplicates for every quantitative experiment using Microsoft Excel. To determine the sensitivity of the model is to its parameters, we employed the Latin Hypercube Sampling method. First, we assumed each parameter is normally distributed, whose mean is the estimated value from the data (either experimentally and numerically derived) and standard deviation is taken to be 1% of the parameter value. Using this method, we selected 200 parameter sets and generated 200 realization curves for the model, which are plotted along with the solution curve. The 80%, 90% and 95% confidence intervals were constructed to contain 80%, 90% and 95% of the realization curves, respectively.

### Model

The mathematical model presented in this manuscript aims to describe key features associated with basal and activated transcription of the HIV-1 genome. In order to produce a model which closely represents the series of events which occur *in vivo*, the associated model parameters have been determined using *in vitro* experiments in HIV-1 infected T-cell (J1.1) and macrophage (U1) cell lines, with the exception of transcription rates from the activated promoter state (α_*m1A*_ and α_*m2A*_), which we obtained by numerical fitting of our model predictions to the experimental measurements of TAR and *env*. Our model, as shown in Fig. 1, categorizes the long-terminal repeat (LTR) into three states; a repressed state (denoted by LTR_*R*_), an intermediate state (denoted by LTR_*I*_), and an activated state (denoted by LTR_*A*_), similar to that used in a previous report^27^. Furthermore, the relative proportions of viral RNAs, including short, non-coding RNA (TAR) and long genomic RNA (*env*), and resulting functional proteins have been attributed to each LTR state.

Activation of the HIV-1 LTR is dependent on Tat association with CDK9 and Cyclin T_1_. The phosphorylation of Histone H1 is directly correlated with exposure of the promoter and transcriptional activation. As such, a Histone H1 kinase assay (see Materials and Methods section) was used to quantitate activation of the LTR at the three transcriptional states (repressed, intermediate, and activated). The repressed and intermediate HIV-1 promoters are considered to be in the ‘*OFF*’ state while the activated HIV-1 promoter is considered to be in the ‘*ON*’ state. Transitions between these states occur with rates *k*_*ON*_ (repressed to intermediate), *k*_*OFF*_ (intermediate to repressed), *k*_*A*_(Tat) (intermediate to active), and *k*_*I*_ (active to intermediate). The intermediate LTR state to an active LTR transition rate is Tat-dependent and has be described as a step-wise function. In this work we have used constant approximation for this parameter due to extremely rapid transition to the active state. There have been several reports of sustained intracellular levels of TAR RNA during antiretroviral treatment which can range from 1 × 10^1^ to 1 × 10^5^ RNA copies *in vivo*^6,44^. Therefore, assuming a 1:1 binding ratio of TAR:Tat, we conservatively estimated the critical Tat value to be equal to 3001 to allow a large enough pool of Tat protein to overcome potential sequestration by TAR RNA^33,35,48^, and thereby allow for efficient Tat-activated transcription. As a result, we propose that these LTR states and associated rates can be expressed as

1 $${{\rm{LTR}}}_{R}\,\mathop{\longrightarrow }\limits^{{k}_{ON}}\,{{\rm{LTR}}}_{I}$$

2 $${{\rm{LTR}}}_{I}\,\mathop{\longrightarrow }\limits^{{k}_{OFF}}\,{{\rm{LTR}}}_{R}$$

3 $${{\rm{LTR}}}_{I}\,\mathop{\longrightarrow }\limits^{{k}_{A}({\rm{Tat}})}\,{{\rm{LTR}}}_{A}$$

4 $${{\rm{LTR}}}_{A}\,\mathop{\longrightarrow }\limits^{{k}_{I}}\,{{\rm{LTR}}}_{I}$$

We have interpreted the three LTR states as proportions of the total LTR count so that LTR_*R*_ + LTR_*I*_ + LTR_*A*_ = 100%. The rates *k*_*ON*_, *k*_*OFF*_, *k*_*A*_(Tat), and *k*_*I*_ have units of inverse time with the change in each LTR state being a (unitless) proportion of the total LTR count.

A key new feature of our model is the inclusion of three different transcriptional states as described by *in vitro* quantitation of two different HIV-1 RNAs – long, genomic RNA transcripts which we have denoted as *env*_*I*_ and *env*_*A*_ (for transcripts that associate with the corresponding promoter states LTR_*I*_ and LTR_*A*_, respectively)–and one short, non-coding RNA sequence which is denoted by TAR. HIV-1 TAR RNA is produced from a virally infected cell regardless of latency or suppressive cART therapy^33,35^. TAR generation occurs from each of the three LTR states (repressed, intermediate and activated) at specified rates. This short RNA sequence is transcribed by all three promoter states with associated rates as indicated by the following biochemical reactions:

5 $${{\rm{LTR}}}_{R}\,\mathop{\longrightarrow }\limits^{{\alpha }_{{m}_{1},R}}\,{{\rm{LTR}}}_{R}+{\rm{TAR}}$$

6 $${{\rm{LTR}}}_{I}\,\mathop{\longrightarrow }\limits^{{\alpha }_{{m}_{1},I}}\,{{\rm{LTR}}}_{I}+{\rm{TAR}}$$

7 $${{\rm{LTR}}}_{A}\,\mathop{\longrightarrow }\limits^{{\alpha }_{{m}_{1},A}}\,{{\rm{LTR}}}_{A}+{\rm{TAR}}$$

During latent infection (i.e. repressed LTR), RNAPII does not produce full length genomic RNA (*env*). Measurement of *env* is representative of the production of full-length genomic RNA which results in the production of functional virions. The biochemical reactions for *env*_*I*_ and *env*_*A*_ are given by

8 $${{\rm{LTR}}}_{I}\,\mathop{\longrightarrow }\limits^{{\alpha }_{m2,I}}\,{{\rm{LTR}}}_{I}+{{\rm{env}}}_{I}$$

9 $${{\rm{LTR}}}_{A}\,\mathop{\longrightarrow }\limits^{{f}_{m2}({\rm{Tat}})}\,{{\rm{LTR}}}_{A}+{{\rm{env}}}_{A}$$

The production of Tat (i.e. translation of mRNA into a functional protein) occurs in our model through the intermediate, *env*_*I*_, long RNA. The production of Tat further enhances activation of the intermediate LTR state, giving a possible gene-activated feedback mechanism, as well as enhances transcription of activated long RNA transcripts *env*_*A*_, giving a transcription-activated feedback mechanism^61^. The expression of transcripts generates more virions that go on to infect more cells and continue the HIV-1 gene regulation. The Tat protein produced from the long, full-length transcript *env*_*I*_ from the intermediate state of the LTR is expressed as

10 $${{\rm{env}}}_{I}\,\mathop{\longrightarrow }\limits^{{\alpha }_{{p}_{1}}}\,{{\rm{env}}}_{I}+{\rm{Tat}}$$

Both long RNA transcripts, *env*_*I*_ and *env*_*A*_, lead to the production of the HIV-1 Gag polyprotein, Pr55, which in turn produces p24. The levels HIV-1 Pr55 and p24 were visualized using Western blot analysis and quantitated using densitometry. Densitometry counts of Pr55 were then utilized to find the rate of Pr55 production (${\alpha }_{{p}_{2}}$) from both *env*_*I*_ and *env*_*A*_. The production of the HIV-1 capsid protein p24 which is cleaved from the gag polyprotein Pr55 at a calculated rate ${\alpha }_{{p}_{3}}$.

Finally, we expressed degradation and exit of viral products into the extracellular space through the parameters ${\gamma }_{{p}_{1}}$. (degradation of Tat), ${\gamma }_{{m}_{1}}$ (degradation/exit rate of TAR), ${\gamma }_{{m}_{2}}$ (degradation/exit rate of *env*_*I*_ and *env*_*A*_), and ${\gamma }_{{p}_{2}}$ (degradation/exit rate of P_24_). As we are primarily interested in the dynamics of these quantities over a short time frame (e.g. several days) simulations were run with the degradation/exit rate rates set to zero for Tat and p24. Conversely, to account for the variability inherent in the measure of RNA via RT-qPCR, we have attempted to estimate the degradation/edit rates of TAR and *env* using a best-fit to the experimental data. Other authors and references therein have defined the burst frequency as the promoter activation rate (defined as *k*_*A*_(Tat) in our model) divided by the transcript degradation rate (in our model ${\gamma }_{{m}_{1}}$ or ${\gamma }_{{m}_{2}}$ are the short and long mRNA transcript degradation/exit rates)^27^ and so with degradation/exit rates set to zero we effectively study the infinite burst frequency limit. The burst size is defined as the basal transcription rate (in our model $\frac{{\alpha }_{{m}_{2},A}}{{v}_{a}}$) divided by the inactivation rate of the promoter (*k*_*I*_ in our model).

The model described in Fig. 1 and characterized by the biochemical reactions described above is written as a coupled system of differential equations that give the evolution of the three LTR states, the basal and activated transcripts TAR, *env*_*I*_ and *env*_*A*_, the trans-activator of transcription, Tat, and the byproducts Pr55 and p24. From a mathematical point of view, our model follows many other efforts to describe various stages of the HIV/AIDS progression using systems of coupled differential equations. Many of these mathematical models have the objective of capturing cell and virus population dynamics on time scales associated with the long-term progression and treatment of HIV-1 and AIDS over years if not decades^62–64^. Others address the dynamics over shorter time scales of hours and/or days with a focus on processes occurring at the cellular level^27,65,66^. Our focus is on the later time scales for which our *in vitro* experiments have been conducted.

The model we consider here is related to that presented by Chavali *et al*., who were interested in describing noise-driven HIV-1 gene expression and promoter activation^27^. Our work is novel and distinct from previous work in that we explore the role of both basal and activated transcription with application to both T-cells and macrophages and that we base our model parameter estimates on our own *in vitro* experiments. We fit a total of four parameters numerically corresponding to genomic and TAR RNA transcription rates from the activated promoter states and their associated degradation/exit rates. Our focus is on a deterministic model; however, we performed sensitivity analyses of our predictions on the various biochemical rates. Numerical solutions of these coupled equations are described in the next section.

The governing differential equations for the time evolution of LTR_*R*_, LTR_*I*_, LTR_*A*_, Tat, TAR, *env*_*I*_, *env*_*A*_, Pr55 and p24 are given below:

11 $$\frac{d}{dt}[{{\rm{LTR}}}_{R}]={k}_{OFF}{{\rm{LTR}}}_{I}-{k}_{ON}{{\rm{LTR}}}_{R}$$

12 $$\frac{d}{dt}[{{\rm{LTR}}}_{I}]=-\,[{k}_{A}({\rm{Tat}})+{k}_{OFF}]{{\rm{LTR}}}_{I}+{k}_{I}LT{R}_{A}+{k}_{ON}{{\rm{LTR}}}_{R}$$

13 $$\frac{d}{dt}[{{\rm{LTR}}}_{A}]={k}_{A}({\rm{Tat}}){{\rm{LTR}}}_{I}-{k}_{I}{{\rm{LTR}}}_{A}$$

14 $$\frac{d}{dt}[{\rm{Tat}}]={\alpha }_{{p}_{1}}{{\rm{env}}}_{I}-{\gamma }_{{p}_{1}}{\rm{Tat}}$$

15 $$\frac{d}{dt}[{\rm{TAR}}]={\alpha }_{{m}_{1},R}{{\rm{LTR}}}_{R}+{\alpha }_{{m}_{1},I}{{\rm{LTR}}}_{I}+{\alpha }_{{m}_{1},A}{{\rm{LTR}}}_{A}-{\gamma }_{{m}_{1}}{\rm{TAR}}$$

16 $$\frac{d}{dt}[{{\rm{env}}}_{I}]={\alpha }_{{m}_{2},I}{{\rm{LTR}}}_{I}-({\gamma }_{m,2}+{\alpha }_{{p}_{1}}+{\alpha }_{{p}_{2}}){{\rm{env}}}_{I}$$

17 $$\frac{d}{dt}[{{\rm{env}}}_{A}]={f}_{{m}_{2}}({\rm{Tat}}){{\rm{LTR}}}_{A}-({\gamma }_{m,2}+{\alpha }_{{p}_{2}}){{\rm{env}}}_{A}$$

18 $$\frac{d}{dt}{P}_{r55}={\alpha }_{{p}_{2}}en{v}_{I}+{\alpha }_{{p}_{2}}en{v}_{A}-{\alpha }_{{p}_{3}}{P}_{r55}$$

19 $$\frac{d}{dt}{p}_{24}={\alpha }_{{p}_{3}}{P}_{r55}-{\gamma }_{{p}_{2}}{p}_{24}$$

In our calculations, for the activated transcription rate, *f*_*m2*_(Tat), we have chosen to use the relatively simple stepwise-defined function

20 $${f}_{{m}_{2}}\,({\rm{Tat}})=\{\begin{array}{c}\frac{{\alpha }_{{m}_{2},A}}{{v}_{a}}\,if\,{\rm{Tat}} < {{\rm{Tat}}}_{crit}\\ {\alpha }_{{m}_{2},A}\,if\,{\rm{Tat}}\ge {{\rm{Tat}}}_{crit}\end{array}$$

which allows a step increase (for *v*_*a*_ > 1 and fixed value of ${\alpha }_{{m}_{2},A}$) in the rate of activated transcription once the Tat concentration exceeds a specified critical value Tat_*crit*_.

The first three of these differential equations (Eqs. 11–13) describe the evolution and transitions that occur between the three LTR promoter states. The dynamics of these three quantities depend on the dynamics of Tat through the activation rate *k*_*A*_(Tat). However, if the coefficient *k*_*A*_(Tat) is constant, then the LTR states are mathematically decoupled from other dynamic variables in our model. Our initial computations have used *k*_*A*_(Tat) as a constant although measured Tat-dependent rates can be easily incorporated. The three states LTR_*R*_, LTR_*I*_ and LTR_*A*_ are defined as proportions of the total LTR count and thus we have LTR_*R*_ + LTR_*I*_ + LTR_*A*_ = 100%. That is, the sum of the three LTR state variables, which represent proportion of the total number of LTR sequences, is preserved. The fourth equation (Eq. 14) gives the rate of change of Tat which is produced by the long transcript *env*_I_ and degrades at rate ${\gamma }_{{p}_{1}}$. The fifth equation (Eq. 15) characterizes the dynamics of the transcript TAR, which is generated at various rates by all three promoter states, LTR_*R*_, LTR_*I*_ and LTR_*A*_. The sixth and seventh equations (Eqs. 16–17) describe the dynamics of intermediate and activated transcripts *env*_*I*_ and *env*_*A*_ which are generated by their counterpart promoter states LTR_*I*_ and LTR_*A*_ and are translated to Tat and Pr55 as well degraded. Finally, the eighth and ninth equations give the rates of change of Pr55 and p24.

In addition to describing the dynamics of the transcription process, we have incorporated into the system of differential equations a means to assess the effectiveness of F07#13, Tat peptide mimetic. We have modeled F07#13’s interaction with the HIV-1 LTR as an increase in the transition from LTR_*A*_ to LTR_*I*_ and simultaneous decrease in the transition from LTR_*I*_ to LTR_*A*_. Thus, taking the effect of F07#13 into account, the rate of transition from LTR_*I*_ to LTR_*A*_ becomes $\frac{1}{{w}_{5}}\,{k}_{A}({\rm{Tat}}){{\rm{LTR}}}_{I}$ and the rate of transition from LTR_*A*_ to LTR_*I*_ becomes *w*_1_*k*_1_*LTR*_*A*_. Here, *w*_1_ and *w*_5_ are constants representing the effect of F07#13 which have value greater or equal to 1 that have been obtained experimentally. These constants have been incorporated into the previously described governing equations as follows:

21 $$\frac{d}{dt}[{{\rm{LTR}}}_{I}]=-[\frac{1}{{w}_{5}}{k}_{A}({\rm{Tat}})+{k}_{OFF}]{{\rm{LTR}}}_{I}+{w}_{1}{k}_{I}LT{R}_{A}+{k}_{ON}{{\rm{LTR}}}_{R}$$

22 $$\frac{d}{dt}[{{\rm{LTR}}}_{A}]=\frac{1}{{w}_{5}}{k}_{A}({\rm{Tat}}){{\rm{LTR}}}_{I}-{w}_{1}{k}_{I}{{\rm{LTR}}}_{A}$$

### Parameter fitting

To account for the uncertainty associated with measurements of TAR and *env* in T-cells and macrophages, a numerical means was used to estimate four parameters: 𝛼_*m1A*_, 𝛼_*m*2𝐴_, 𝛾_*m*1_ and 𝛾_*m2*_. The model was fit simultaneously to the experimental values of TAR and total *env* (e.g. time series data of TAR and *env* performed in three biological replicate and analyzed in technical triplicate by RT-qPCR) independently for T-cells and Macrophages. In order to initialize the parameter estimation process, experimentally derived rates for the production of TAR and *env* were used as initial approximations of the parameter values. The initial approximations of the degradation/exit rates for TAR and *env* are taken as the linear rates of change of extracellular TAR and *env* RNA. The objective function to be minimized is the standard objective in least squared fitting. All time series data was simultaneously fit for each triplicates and replicates of both env and TAR. The function fmincon – a commercial product of MATLAB – was used to estimate these parameters within reasonable ranges. The ranges for the estimation extend from 0.1 to 10 times the initial approximations. Through testing, changing this range does not affect the overall results. The estimated values are given in Table 1.

### Impact statement

We propose a novel 3-state LTR model (LTR_*R*_, LTR_*I*_, LTR_*A*_) incorporating long and short RNAs that offers insight into HIV-1 transcriptional dynamics in latently infected macrophages and T-cells and provides a framework for future mathematical analysis of HIV-1 transcriptional interactions and drug interventions. Specifically, presence of short non-coding RNAs such as TAR at high levels such as that produced from an activated LTR state may explain Tat sequestration and transcription inactivation leading to viral latency.

## Supplementary information

Supplementary Information.

## Data Availability

The datasets generated during and/or analyzed during the current study are available from the corresponding author on reasonable request.
