# Supplementary material for: Differences in Transcriptional Dynamics Between T-cells and Macrophages as Determined by a Three-State Mathematical Model
Source: Sci Rep. 2020 Feb 10;10:2227. doi: 10.1038/s41598-020-59008-0 (PMC7010665; doi:10.1038/s41598-020-59008-0)
Supplement: Supplementary file 1 — Supplementary Information. [file 41598_2020_59008_MOESM1_ESM.pdf]

Supplementary Information for

Differences in Transcriptional Dynamics Between T-cells and Macrophages as Determined by a Three-State Mathematical Model

Catherine DeMarino, Maria Cowen, Michelle L. Pleet, Daniel O. Pinto, Pooja Khatkar, James Erickson, Steffen S. Docken, Nicholas Russell, Blake Reichmuth, Tin Phan, Yang Kuang, Daniel M. Anderson, Maria Emelianenko, Fatah Kashanchi

Correspondence: Fatah Kashanchi, Ph.D., Laboratory of Molecular Virology, George Mason University, Discovery Hall Room 182, 10900 University Blvd., Manassas, VA 20110, USA, Tel.: 703-993-9160, Fax: 703-993-7022, Email: fkashanc@gmu.edu; Daniel Anderson, Professor, Mathematical Sciences, George Mason University, MS 3F2, 4400 University Dr, Fairfax VA 22030, USA, Tel: 703-993-1482, Email: danders1@gmu.edu; Maria Emelianenko, Professor, Mathematical Sciences, George Mason University, MS 3F2, 4400 University Dr, Fairfax VA 22030, USA, Tel: 703-993-9688, Email: memelian@gmu.edu

This PDF file includes:

Figs. S1 to S5

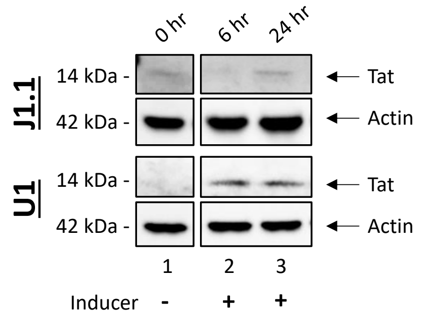

Supplemental Figure 1. HIV-1 Tat is Increased Post-treatment with an Inducer. J1 (HIV-1 infected T-cell) and U1 (HIV-1 infected myeloids) were placed in low serum media (0.1% FBS) for 36 h, and subsequently incubated in 20% FBS media and treated with an inducer (IR). Whole cell extracts were then analyzed using Western blot for the presence of Tat protein. Western blots were analyzed by densitometry, counts were normalized and used to construct parameters for mathematical modeling to assess relative change in the abundance of Tat protein.

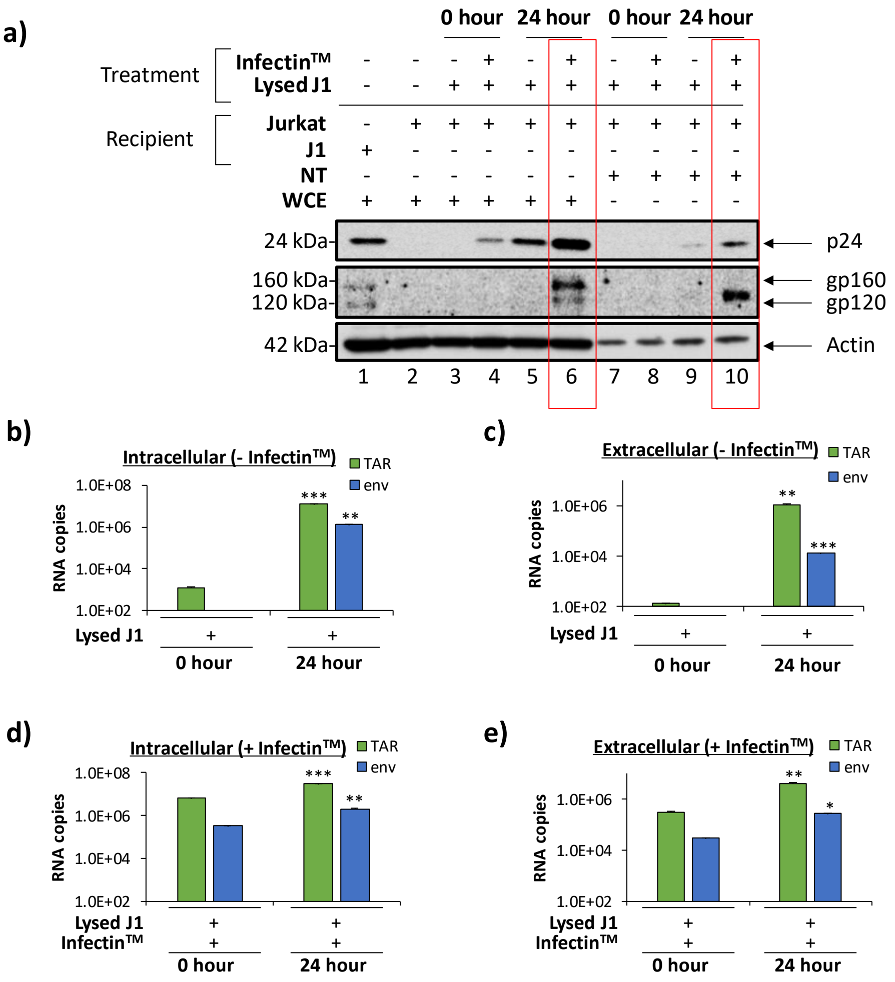

Supplemental Figure 2. Timed infection of uninfected T-cells. HIV infected J1 cells and uninfected Jurkat T-cells were cultured. J1 cells were serum starved in media containing 0.1% FBS for 3 days, followed by activation in media containing 20% FBS media for the following time points: 0 and 24 hours. Intracellular material was isolated using non-detergent lysis buffer and added to uninfected Jurkat cells ± Infectin^{TM} for 2 days. **A)** Jurkat cells were lysed, while the supernatants were enriched using NT80/82/86 beads, both were run on an SDS-PAGE gel and analyzed for the presence of HIV-1 markers and actin. Intracellular **(B)** and extracellular **(C)** RNA was from the recipient cells treated with J1 whole cell extract were analyzed by RT-qPCR for the presence of TAR and *env* RNA. Intracellular **(D)** and extracellular **(E)** RNA was from the recipient cells treated with J1 whole cell extract were analyzed by RT-qPCR for the presence of TAR and *env* RNA. For the isolation of extracellular RNA, supernatants were enriched using NT80/82/86 particles.

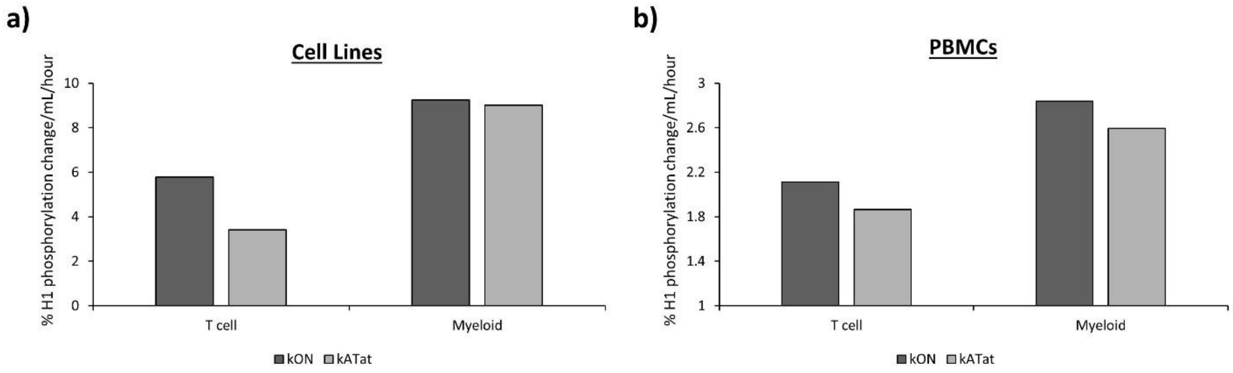

Supplemental Figure 3. Comparison of LTR Activation Rates Between T-cell and Myeloid Cell Lines and Primary Cells. Cell line (T-cell and myeloid; panel **A**) and primary T-cell and myeloid cells (PBMCs; panel **B**) k_{ON} and k_ATat rates were calculated using densitometry counts of CDK9-IP Histone H_1 *in vitro* kinase images. Percent of H_1 phosphorylation change/mL/hour was calculated and graphed.

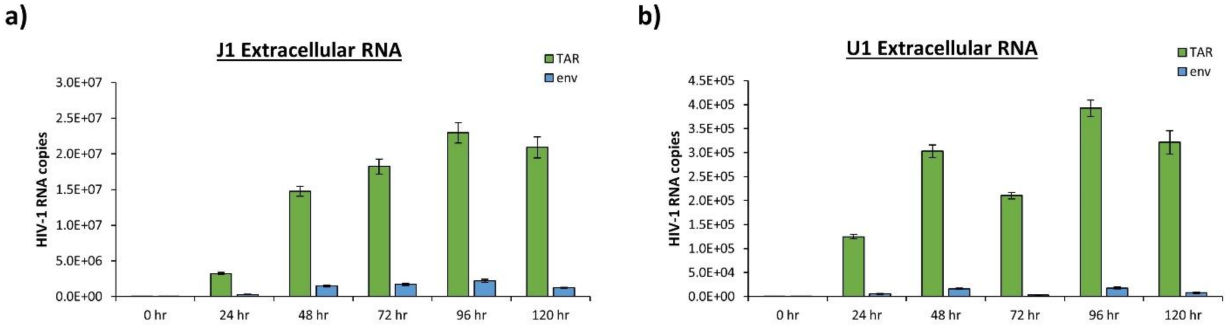

Supplemental Figure 4. EV-associated TAR and *env* RNA Increase Over Time. J1 (HIV-1 infected T-cell) and U1 (HIV-1 infected myeloids) were placed in low serum media (0.1% FBS) for 36 h, and subsequently incubated in 20% FBS media for 0, 24, 48, 72, 96, and 120 hr in biological triplicate. EVs were isolated from conditioned cell culture supernatant using NT80/82. Total EV RNA was isolated and was analyzed in technical triplicate by RT-qPCR for the presence of TAR and *env* RNA.

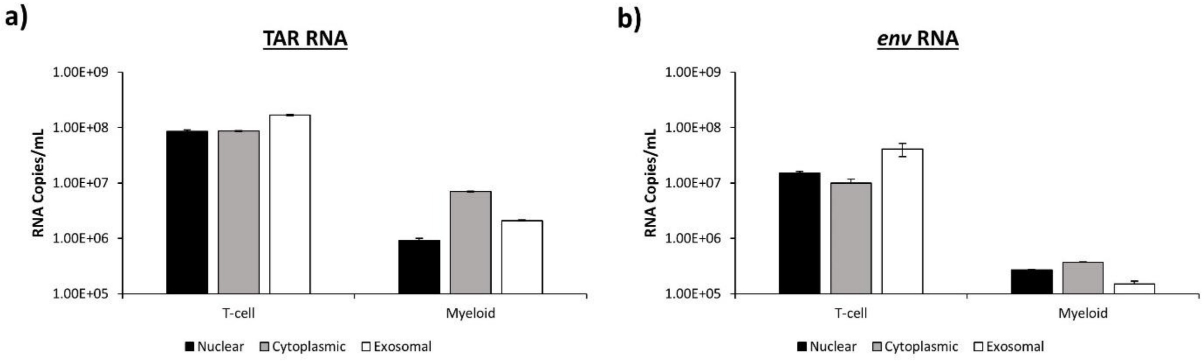

Supplemental Figure 5. Differences in the Distribution of Viral RNAs Within T-cell and Myeloids. T-cell (J1) and myeloids (U1) were cultured and nuclear and cytoplasmic extracts were isolated and analyzed alongside RNA isolated from exosomes by RT-qPCR for the presence of TAR and *env* (full length) RNA. Bars indicate an average of three technical replicates ± S.D
